# Similar predictive performance and clinical utility of the Kidney Failure Risk Equation using EKFC or CKD-EPI estimated glomerular filtration rate

**DOI:** 10.1093/ckj/sfag187

**Published:** 2026-06-08

**Authors:** Antoine Créon, Malou Magnani, Carolien C H M Maas, Merel van Diepen, Aurora Caldinelli, William A Russel, Friedo W Dekker, Juan-Jesus Carrero, Edouard L Fu

**Affiliations:** Department of Medical Epidemiology and Biostatistics, Karolinska Institutet, Stockholm, Sweden; Department of Medical Epidemiology and Biostatistics, Karolinska Institutet, Stockholm, Sweden; Department of Clinical Epidemiology, Leiden University Medical Center, Leiden, The Netherlands; Department of Medical Epidemiology and Biostatistics, Karolinska Institutet, Stockholm, Sweden; Department of Clinical Epidemiology, Leiden University Medical Center, Leiden, The Netherlands; Department of Clinical Epidemiology, Leiden University Medical Center, Leiden, The Netherlands; Department of Medical Epidemiology and Biostatistics, Karolinska Institutet, Stockholm, Sweden; Department of Medical Epidemiology and Biostatistics, Karolinska Institutet, Stockholm, Sweden; Department of Clinical Epidemiology, Leiden University Medical Center, Leiden, The Netherlands; Department of Medical Epidemiology and Biostatistics, Karolinska Institutet, Stockholm, Sweden; Division of Nephrology, Department of Clinical Sciences, Danderyd Hospital, Danderyd, Sweden; Department of Medical Epidemiology and Biostatistics, Karolinska Institutet, Stockholm, Sweden; Department of Clinical Epidemiology, Leiden University Medical Center, Leiden, The Netherlands

**Keywords:** CKD, CKD-EPI equation, cystatin C, EKFC equation, KFRE equation

## Abstract

**Background:**

International guidelines recommend the Kidney Failure Risk Equation (KFRE), which includes estimated glomerular filtration rate (eGFR), to guide nephrology referral and preparation for kidney replacement therapy in chronic kidney disease (CKD). The European Kidney Function Consortium (EKFC) eGFR equations are increasingly recognized as alternatives to CKD-EPI, but their impact on KFRE predictive performance and clinical utility is unknown.

**Methods:**

We identified adults with same-day creatinine and cystatin C measurements and an albuminuria assessment in Stockholm between 2011 and 2021. We evaluated 2- and 5-year 4-variable KFRE performance using CKD-EPI or EKFC eGFR equations based on creatinine (eGFR_cr_), cystatin C (eGFR_cys_), or both filtration markers (eGFR_cr-cys_). Discrimination was assessed using time-dependent area under the curve (AUC), calibration with calibration plots, and overall accuracy with predicted risk distributions and Brier scores. Clinical utility was evaluated using decision curve analysis (DCA) at guideline-recommended thresholds. All analyses accounted for the competing risk of death.

**Results:**

Among 27 125 participants (median age 75 years; 45% women), KFRE discrimination was consistently excellent across all equations, filtration markers, and prediction horizons (AUC 0.95–0.97). For eGFR_cr_, EKFC and CKD-EPI showed similar calibration at the 2- and 5-year horizons. However, calibration was better for EKFC-based predictions than CKD-EPI when using eGFR_cys_ or eGFR_cr-cys_. DCA demonstrated nearly identical clinical utility for CKD-EPI and EKFC at guideline-recommended thresholds.

**Conclusions:**

In this North-European health system study, estimating eGFR with EKFC or CKD-EPI equations does not materially alter the predictive performance or clinical utility of the KFRE.

## Graphical Abstract

**Graphical Abstract:**
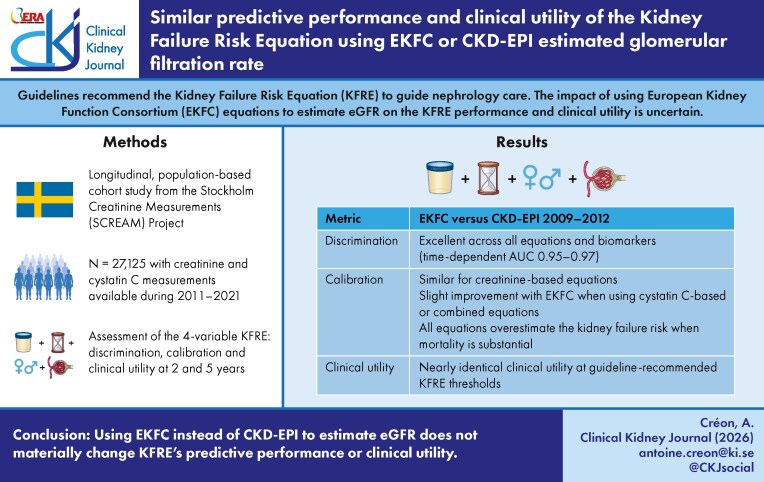

KEY LEARNING POINTS **What was known:**

- The Kidney Failure Risk Equation (KFRE), incorporating estimated glomerular filtration rate (eGFR), is recommended to guide nephrology care, but its performance has mainly been evaluated using CKD-EPI equations.
- EKFC equations may provide more accurate eGFR estimates in European populations, yet their impact on KFRE discrimination, calibration, and clinical utility was uncertain.

**This study adds:**

- In a large European cohort, replacing CKD-EPI with EKFC equations does not materially change KFRE discrimination or clinical utility for predicting 2- and 5-year kidney failure risk.
- EKFC equations marginally improve KFRE calibration when using cystatin C or combined creatinine–cystatin C eGFR, while performance using creatinine-based eGFR remains comparable between equations.
- Accounting for the competing risk of death suggests that the KFRE may overestimate absolute kidney failure risk when mortality risk is substantial.

**Potential impact:**

- Estimating eGFR with EKFC or CKD-EPI equations does not materially alter the predictive performance or clinical utility of the KFRE.
- When mortality risk is substantial, clinicians should consider that KFRE may overestimate absolute kidney failure risk when communicating risk to patients.

## INTRODUCTION

Accurate prediction of kidney failure risk is central to optimizing care for patients with chronic kidney disease (CKD). International guidelines recommend the Kidney Failure Risk Equation (KFRE) to guide nephrology referral and preparation for kidney replacement therapy (KRT) among individuals with an estimated glomerular filtration rate (eGFR) between 10 and 60 ml/min/1.73 m² [1]. In its four-variable version, the KFRE combines age, sex, eGFR, and albuminuria to estimate an individual’s 2- and 5-year risk of kidney failure [2]. Originally developed in North-America using the creatinine-based Chronic Kidney Disease Epidemiology Collaboration 2009 equation (CKD-EPI) [3], the KFRE has since been validated in diverse populations [4].

The KDIGO 2024 guidelines emphasize that eGFR equations should, whenever possible, be tailored to the geographical context and chosen to best approximate measured GFR in the populations in which they are applied [1]. In Europe, some studies have reported reduced bias and improved accuracy of the European Kidney Function Consortium (EKFC) equations [5, 6] compared with CKD-EPI, especially among younger and older adults [7, 8, 9]. These potential performance gains, together with the applicability of EKFC equations to both children and adults, have motivated proposals to transition from CKD-EPI to EKFC at the continental level [10].

The choice of eGFR equation could meaningfully shift KFRE risk estimates, as EKFC can yield values that differ from CKD–EPI by a median of ∼5 ml/min/1.73 m² in populations and by as much as 25 ml/min/1.73 m² in some individuals [11]. KFRE can be calculated using eGFR estimated from creatinine (eGFR_cr_), cystatin C (eGFR_cys_), or both (eGFR_cr-cys_), with cystatin C being increasingly recommended when creatinine may be unreliable [1]. Understanding how the KFRE performs when paired with EKFC equations across these filtration markers is therefore essential, as changes in eGFR inputs could influence decisions about referral, multidisciplinary care, and KRT planning.

We therefore aimed to evaluate how replacing CKD-EPI with EKFC equations to estimate eGFR would affect the predictive performance and clinical utility of the KFRE in a European setting.

## MATERIALS AND METHODS

We followed the Transparent Reporting of a multivariable prediction model for Individual Prognosis Or Diagnosis (TRIPOD) statement for reporting [12].

### Data source

We used data from the Stockholm CREAtinine Measurement (SCREAM) project, which is a healthcare utilization cohort of individuals residing or accessing healthcare in the region of Stockholm, Sweden [13]. SCREAM contains longitudinal healthcare information from more than 3 million Stockholm residents who underwent creatinine assessments between January 2006 and December 2021. Through linkage across national and regional databases, the dataset contains data on demographics, laboratory tests, diagnoses, vital status, dispensed medication prescriptions, and healthcare utilization, with virtually no loss to follow-up [13]. Data on dialysis and kidney transplantation were obtained from the Swedish Renal Registry, a nationwide registry with complete coverage of kidney failure with replacement therapy (KFRT) cases, and data on mortality were obtained from the Swedish Cause of Death Registry [14]. The Regional Ethical Review Board in Stockholm approved the study (reference 2017/793-31). Informed consent was not required, as all data were de-identified by the Swedish Board of Health and Welfare.

### Study design and patient selection

We identified all cystatin C measurements taken in the outpatient setting between 1 January 2011 and 31 December 2021. We excluded measurements before 2011 since national standardization of cystatin C measurements occurred in Sweden in 2010 [15]. Additionally, we required the presence of a creatinine measurement on the same day, and an albuminuria/proteinuria measurement within 12 months before or after the creatinine/cystatin C measurement. We selected the albuminuria measurement closest to the date of creatinine/cystatin C measurement, and then converted urine protein-to-creatinine ratio and dipstick proteinuria measurements to albumin-to-creatinine ratio (UACR) using the validated adjusted equation by Sumida *et al*., which includes sex, hypertension, and diabetes [16].

eGFR measurements were considered eligible if the patient was aged ≥18, eGFR was 10-60 ml/min/1.73 m^2^ calculated with the eGFR_cr_ CKD-EPI 2009 without race coefficient, and the patient was not receiving dialysis or a kidney transplant recipient (Fig. S1). Eligibility was defined using the eGFR_cr_ CKD-EPI 2009 equation as it is currently recommended in Europe and to maintain consistency with the KFRE derivation cohort [2, 17, 18]. If a patient had more than one eligible eGFR-albuminuria measurement pair, then we selected one at random. We defined the index date as the most recent date between the selected eGFR and albuminuria measurements to prevent immortal time. For each patient, the model prediction origin (the point from which the model made its predictions) was the index date, corresponding to the moment a clinician had information on both eGFR and albuminuria results to calculate KFRE, regardless of how long the patient has had CKD, mirroring real-world practice in which patients enter care at different stages.

### Study predictors

Predictors used in the non-North American, four-variable KFRE were age, sex, eGFR measured in ml/min/1.73 m^2^, and UACR measured in mg/g. We calculated the eGFR with the creatinine or cystatin C-based EKFC or CKD-EPI 2009–2012 equations (Table S1). In this manuscript, we use eGFR_cr_, eGFR_cys_, and eGFR_cr-cys_ to denote equations based on creatinine, cystatin C, or both markers, respectively, and specify CKD-EPI or EKFC to indicate the corresponding estimating equation. Throughout, CKD-EPI refers exclusively to the 2009–2012 equations which are currently recommended in Europe, and not to the race-free 2021 updates [17, 18].

### Study outcomes

Study outcomes were 2-year and 5-year risk of KFRT, defined as the composite of dialysis initiation or kidney transplantation, in line with the definition used in the original KFRE development study [2]. Patients were followed from the index date until the occurrence of KFRT, death, migration from Stockholm region, or administrative censoring (31st December 2021), whichever occurred first.

### Study covariates

For each patient, we extracted a number of baseline characteristics, including age, sex, UACR, comorbidities, and medication use, all detailed in Table S2. We identified comorbidities based on recorded clinical diagnoses. Medications were ascertained through filled prescriptions at Swedish pharmacies using the Prescribed Drug Registry [19], with a medication considered ongoing if dispensed within 180 days prior to the index date.

### Subgroup and sensitivity analyses

We conducted subgroup analyses stratified by sex, age group (<75 vs. >75 years), and KDIGO CKD stage (G3 vs. G4-5) to evaluate the consistency of our findings across clinically relevant patient groups. Additionally, we assessed consistency of results in the subgroup of patients with a preceding eGFR <60 ml/min/1.73 m² at least 3 months before the eGFR measurement used to define eligibility (i.e. confirmed CKD).

### Statistical analysis

We assessed the predictive performance of the 2-year and 5-year KFRE with each equation group (EKFC and CKD-EPI) and filtration marker (eGFR_cr_, eGFR_cys_, and eGFR_cr-cys_) across four domains: discrimination, calibration, overall accuracy, and clinical utility, reporting all metrics recommended by the STRengthening Analytical Thinking for Observational Studies (STRATOS) initiative [20]. For each filtration marker, the corresponding CKD-EPI equation was used as the reference. Although the KFRE was not developed using competing risk methods, in our study, we considered death a competing event to validate how well the KFRE predicts the absolute risk of KFRT [21]. In the main text, we explain these metrics in simple terms, while technical details on their calculation and interpretation are provided in the Supplemental Methods and Table S3.

### Discrimination

Discrimination measures how well the model distinguishes between individuals who will develop KFRT and those who will not. We assessed discrimination using the time-dependent area under the receiver operating characteristic curve [time-dependent area under the curve (AUC)] [22, 23], which accounts for the competing risk of death through the way cases and controls are defined. An AUC of 1 indicates perfect discrimination, while an AUC of 0.5 reflects no better than chance.

### Calibration

Calibration describes how closely the predicted risks from the model match the actual observed risks at the relevant time horizon. The primary assessment of calibration is the calibration plot, while complementary numerical metrics, such as the calibration intercept, calibration slope, and the observed-to-expected (O/E) ratio provide additional quantitative indices [24]. Calibration plots provide a visual comparison between predicted and observed risks. The calibration intercept reflects systematic over- or underestimation, while the calibration slope indicates whether predicted risks are too extreme or too moderate compared to observed outcomes. Finally, the O/E ratio compares the observed proportion of events to the average predicted risk.

### Overall accuracy

Overall accuracy summarizes the model’s performance by combining calibration and discrimination. We assessed this using two approaches: (i) the distribution of predicted 2- and 5-year risks of KFRT among individuals who did and did not develop KFRT, and (ii) summary measures based on the Brier score [20]. The distribution of predicted risks provides an intuitive assessment of how well the model separates high-risk from low-risk individuals. The Brier score is the squared difference between predicted risks and observed outcomes at a given time horizon, with lower values indicating better overall performance. The scaled Brier score expresses this value relative to a non-informative model, i.e. a model assigning average observed risk to all individuals, with positive values indicating improvement over the non-informative model and higher values indicating better overall performance. Finally, the delta scaled Brier score measures the difference in performance between two models.

### Clinical utility

Clinical utility assesses whether using a prediction model leads to better clinical decisions. We assessed this using decision curve analysis (DCA) [25]. DCA estimates net benefit across clinically relevant thresholds, weighing the benefit of correctly identifying high-risk patients against the harm of unnecessary interventions.

For the 2-year KFRE, we investigated net benefit at risk thresholds of 10% and 40%, which are currently recommended by KDIGO to initiate multidisciplinary care and preparation for KRT, respectively [1]. This analysis was restricted to patients with an eGFR_cr_ CKD-EPI of 10–29 ml/min/1.73 m². For the 5-year KFRE, we calculated net benefit at risk thresholds of 3%–5% among patients with an eGFR_cr_ CKD-EPI of 30–59 ml/min/1.73 m², as referral to nephrology is recommended when the predicted risk exceeds these thresholds [1].

Because albuminuria, creatinine, and cystatin C measurements were required for inclusion, there were no missing data in this study. All analyses were conducted using R version 4.5.1. The code is available at https://github.com/CareMeds/KFRE-performance-for-EKFC-vs-CKD-EPI.

## RESULTS

### Characteristics of the population

A total of 191 720 individuals had at least one cystatin C measurement between January 2011 and December 2021, of whom 84 923 also had a creatinine measurement on the same day and an albuminuria assessment within 12 months. We excluded 50 200 patients with an eGFR <10 or ≥60 ml/min/1.73 m². After applying all exclusion criteria, 27 125 patients remained for analysis (Fig. S2).

The median age was 75 years, 44.8% were women, and the median eGFR_cr_ CKD-EPI was 45 ml/min/1.73 m² (Table 1). The most frequently diagnosed comorbidities were hypertension (81%) and diabetes mellitus (37%), while the most prescribed medications were angiotensin-converting enzyme (ACE) inhibitors or angiotensin 2 receptor blockers (ARBs) (65%) and beta-blockers (52%). When comparing equations, eGFR_cr_ values calculated with EKFC were generally lower than eGFR_cr_ values calculated with CKD-EPI. However, eGFR_cys_ and eGFR_cr-cys_ values tended to be greater when calculated with EKFC than with CKD-EPI (Fig. S3).

**Table 1:** Baseline characteristics of patients with simultaneous creatinine and cystatin C measurements (2011–2021) and CKD-EPI eGFR_cr_ 10–60 ml/min/1.73 m² in the SCREAM cohort.

Characteristic	*N* = 27 125
Age, median (IQR), yr	75 (67, 82)
Female, *n*(%)	12 139 (45%)
eGFR category, *n*(%)	
G3a (45 to <60)	13 590 (50%)
G3b (30 to <45)	8 257 (30%)
G4 (15 to <30)	4 406 (16%)
G5 (10 to <15)	872 (3%)
Median eGFR (IQR), ml/min/1.73 m^2^	
CKD-EPI_cr_ 2009	45 (33, 53)
CKD-EPI_cys_ 2012	37 (25, 49)
CKD-EPI_cr-cys_ 2012	41 (29, 51)
EKFC_cr_	42 (32, 50)
EKFC_cys_	40 (29, 52)
EKFC_cr-cys_	42 (31, 50)
Median UACR (IQR), mg/g	22 (15, 88)
Comorbidities, *n*(%)	
Myocardial infarction	3676 (13%)
Other ischemic heart disease	7071 (26%)
Hypertension	22 010 (81%)
Heart failure	6637 (24%)
Stroke	3374 (12%)
Other cerebrovascular disease	3636 (13%)
Arrhythmia	8112 (29%)
Peripheral vascular disease	2723 (10%)
Diabetes mellitus	9993 (36%)
Cancer in previous year	3390 (12%)
Chronic obstructive pulmonary disease	3127 (11%)
Liver disease	1233 (4%)
Medications, *n*(%)	
Beta blocker	14 220 (52%)
Calcium channel blocker	7271 (26%)
Diabetes medications	10 259 (37%)
Diuretic	12 233 (45%)
ACEi/ARB	17 752 (65%)
Lipid lowering drug	11 716 (43%)
NSAID	2855 (10%)

Continuous variables are described as median (Q1–Q3) and categorical variables as *n*(%). eGFR, estimated glomerular filtration rate (creatinine-based CKD-EPI 2009 equation); UACR, urinary albumin-to-creatinine ratio; ACEi, angiotensin-converting enzyme inhibitor; ARB, angiotensin 2 receptor blocker; and NSAID, non-steroidal anti-inflammatory drug.

At 2 years, a total of 620 (2.3%) patients had experienced KFRT, and 4214 patients (15.5%) had died without KFRT (Table S4). At 5 years, KFRT had occurred in 1265 patients (4.7%) and 8287 patients (30.6%) had died without KFRT. A comparison with the non-North American KFRE development cohort is provided in Table S5.

### Discrimination

**Figure 1:**
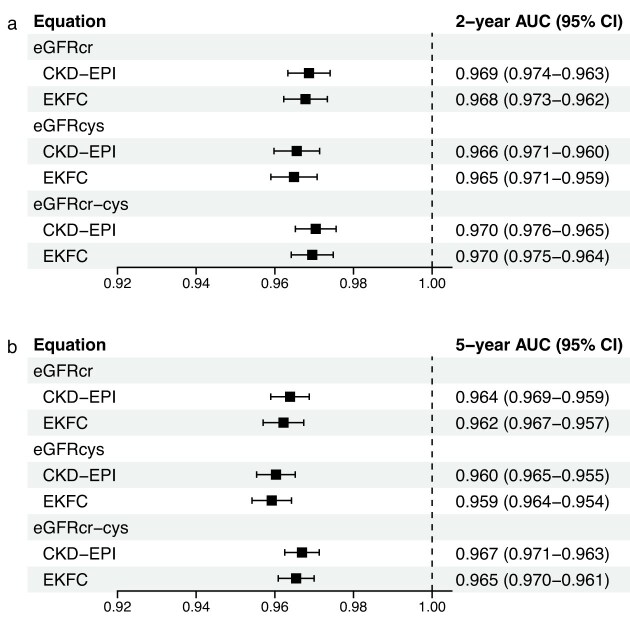
Discrimination of the 2-year and 5-year KFRE predictions using CKD-EPI or EKFC eGFR equations. Discrimination was assessed by the time-dependent area under the receiver operating characteristics curve (AUC), with higher values indicating better ability to distinguish between individuals who did and did not progress to KFRT at the prediction horizon. Panel (a) represents the 2-year AUC and panel (b) the 5-year AUC, for the 2-year and 5-year KFRE predictions, respectively. Error bars represent 95% confidence intervals.

There were no meaningful differences in discrimination between KFRE models calculated with CKD-EPI and EKFC equations across filtration markers or prediction horizons. Both the 2-year and 5-year KFRE models demonstrated consistently high time-dependent AUC values ranging from 0.95 to 0.97 (Fig. 1).

### Calibration

**Figure 2:**
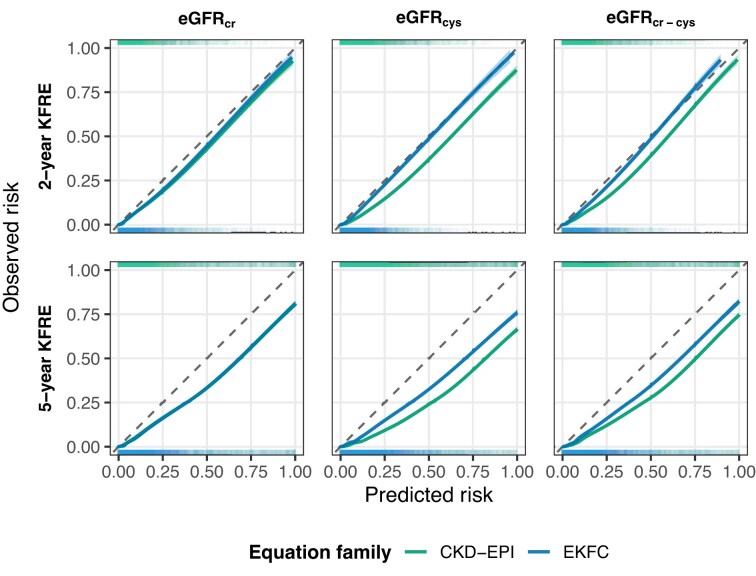
Calibration plots of 2-year and 5-year KFRE using CKD-EPI or EKFC eGFR equations. Observed risks (*y*-axis) are plotted against predicted risks (*x*-axis) for each equation and filtration marker combination. The solid lines represent loess-smoothed calibration curves, and the dashed diagonal line denotes perfect calibration (observed = predicted). Rug plots along the axes illustrate the distribution of predicted risks. Shaded areas represent 95% confidence intervals. Panels are organized by prediction horizon (2-year top row, 5-year bottom row) and eGFR estimation method: creatinine-based (left column), cystatin C–based (middle column), and combined creatinine–cystatin C (right column).

For the 2-year KFRE using eGFR_cr_, both EKFC and CKD-EPI showed good agreement between predicted and observed risks: calibration curves were slightly below the identity line, calibration intercepts were near 0 and calibration slopes close to 1.2 with confidence intervals excluding 1 (Fig. 2, Table 2). EKFC demonstrated superior calibration compared with CKD-EPI when using eGFR_cys_ and eGFR_cr-cys_, as demonstrated by calibration curves closer to the identity line and calibration intercepts closer to 0 (eGFR_cys_: 0.00 [−0.08;0.09] vs. −0.40 [−0.48; −0.32]; eGFR_cr-cys_: 0.08 [−0.01;0.16] vs. −0.23 [−0.32; −0.15] for EKFC and CKD-EPI, respectively) (Fig. 2, Table 2).

**Table 2:** Calibration intercept and calibration slope for the 2-year and 5-year KFRE predictions using CKD-EPI or EKFC eGFR equations.

	2-year KFRE		5-year KFRE	
Equation	Calibration intercept	Calibration slope	Calibration intercept	Calibration slope
**eGFR_cr_**				
CKD-EPI	−0.07 (−0.15; 0.02)	1.17 (1.06; 1.27)	−0.58 (−0.65; −0.52)	0.98 (0.93; 1.04)
EKFC	−0.02 (−0.11; 0.06)	1.21 (1.10; 1.31)	−0.57 (−0.64; −0.51)	1.01 (0.96; 1.07)
**eGFR_cys_**				
CKD-EPI	−0.40 (−0.48; −0.32)	1.26 (1.15; 1.37)	−1.02 (−1.08; −0.96)	1.04 (0.98; 1.10)
EKFC	0.00 (−0.08; 0.09)	1.23 (1.13; 1.34)	−0.62 (−0.68; −0.56)	1.01 (0.95; 1.07)
**eGFR_cr-cys_**				
CKD-EPI	−0.23 (−0.32; −0.15)	1.27 (1.16; 1.39)	−0.81 (−0.87; −0.74)	1.04 (0.99; 1.10)
EKFC	0.08 (−0.01; 0.16)	1.28 (1.17; 1.39)	−0.51 (−0.58; −0.45)	1.04 (0.98; 1.10)

Calibration intercepts reflect overall under- or over-prediction (ideal value = 0), and calibration slopes quantify whether a model is systematically under or over-confident in the risks it assigns (ideal value = 1). Estimates are shown with 95% confidence intervals.

For the 5-year KFRE, both EKFC and CKD-EPI overestimated risk across filtration markers (eGFR_cr_, eGFR_cys_, or eGFR_cr-cys_): calibration curves were consistently below the identity line, while calibration intercepts were negative with confidence intervals excluding 0. EKFC and CKD-EPI performed similarly for eGFR_cr_, but EKFC showed better calibration than CKD-EPI for eGFR_cys_ and eGFR_cr-cys_ (calibration intercept eGFR_cys_: −0.62 [−0.68; −0.56] vs. −1.02 [−1.08; −0.96]; eGFR_cr-cys_: −0.51 [−0.58; −0.45] vs. −0.81 [−0.87; −0.74] for EKFC and CKD-EPI, respectively) (Fig. 2, Table 2).

O/E ratios supported these findings, indicating risk overestimation at 5 years and, for CKD-EPI, also at 2 years when using eGFR_cys_ or eGFR_cr-cys_ (Fig. S4).

### Overall accuracy

Across all eGFR equations, predicted risks among individuals who did not experience KFRT or died were tightly concentrated at very low values, whereas those who progressed to KFRT exhibited broader distributions extending into higher risk ranges, a pattern consistent at both the 2-year and 5-year prediction horizons (Fig. S5). Relative to CKD-EPI, EKFC was associated with small shifts in predicted risks that mirrored underlying eGFR distribution differences, with EKFC-based eGFR_cys_ and eGFR_cr-cys_ yielding slightly lower risk estimates at both prediction horizons. Individual-level changes were typically on the order of 0.1% and were consistent with these population-level patterns (Fig. S6).e

Brier score-based metrics revealed minimal differences between the EKFC and CKD-EPI equations across filtration markers and horizons: for the 2-year prediction horizon, CKD-EPI-based KFRE showed slightly better overall accuracy than EKFC, as reflected by lower Brier scores, higher scaled Brier scores, and negative delta scaled Brier values (Table S6). For the 5-year prediction horizon, this pattern persisted for eGFR_cr_ but reversed for the other filtration markers: EKFC equations achieved slightly better overall accuracy as reflected by lower Brier scores, higher scaled Brier scores, and positive delta scaled Brier values (Table S6).

### Clinical utility

**Figure 3:**
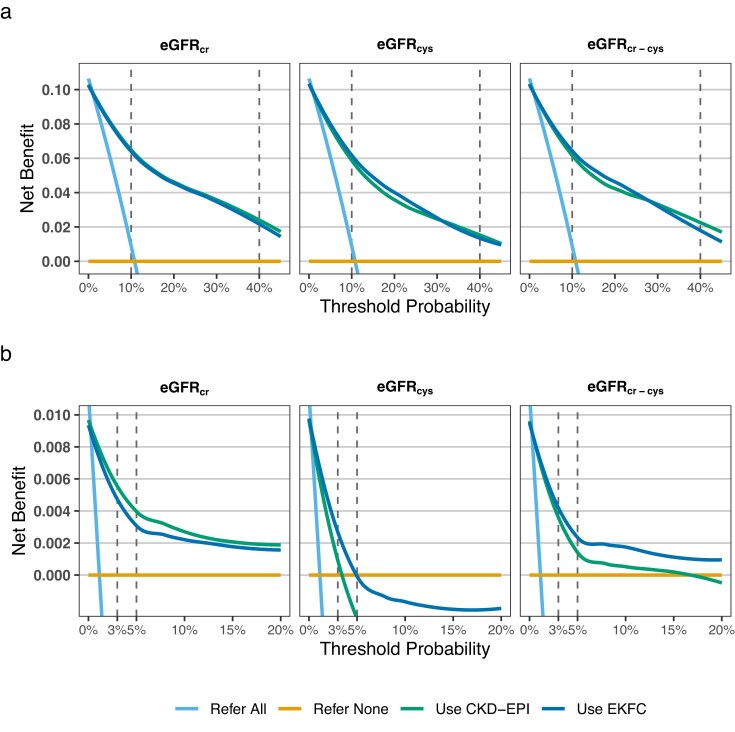
DCA of 2-year and 5-year KFRE predictions using CKD-EPI or EKFC eGFR equations. Panel (a) shows the 2-year KFRE in the subset of patients with eGFRcr CKD-EPI 10–29 ml/min/1.73 m^2^, and panel (b) shows the 5-year KFRE in the subset of patients with eGFRcr CKD-EPI 30–59 ml/min/1.73 m^2^. Net benefit is plotted across a range of threshold probabilities, with higher curves indicating greater clinical utility. The vertical dashed lines represent the currently recommended thresholds to guide nephrology referral (5-year KFRE of 3%–5%), initiation of multidisciplinary care (2-year KFRE of 10%), and preparation for KRT (2-year KFRE of 40%). The “refer all” and “refer none” strategies are included as references. Note that the *y*-axis scale in panel (b) is 10-fold smaller than in panel (a).

For the 2-year KFRE, risks estimated from EKFC and CKD-EPI equations both produced high and nearly identical net benefit across filtration markers and across the range of threshold probabilities, including at the currently recommended 10% and 40% thresholds to guide initiation of multidisciplinary care and preparation for KRT, respectively (Fig. 3). For example, using a KFRE threshold of 10% with eGFR_cr_ to guide multidisciplinary care yielded an identical net benefit of 0.065 for both EKFC and CKD-EPI, corresponding to 65 additional true positive identifications of patients who progress to KFRT per 1000 patients, without an increase in unnecessary multidisciplinary care, compared with a strategy of never initiating this care.

For the 5-year KFRE, risks estimated from EKFC and CKD-EPI equations again showed similar net benefit across filtration markers, but the magnitude of benefit was smaller than for the 2-year model. Importantly, eGFR_cys_ estimates offered little to no net benefit: at the currently recommended 3% threshold, only the EKFC equation yielded a meaningful improvement over a strategy of referring no one, whereas at 5%, none of EKFC or CKD-EPI provided net benefit (Fig. 3).v

### Subgroup and sensitivity analyses

KFRE performance patterns were similar with EKFC and CKD-EPI eGFR across sex, age, and CKD stage subgroups. Regardless of the eGFR equation used, discrimination was slightly lower in CKD stages 4 and 5, and risk was overestimated in individuals aged ≥75 years. The use of EKFC eGFR_cys_ modestly underestimated risk in women at higher predicted risk, and net benefit was higher in men and in individuals aged <75 years (Table S7, Figs S7–S11).

KFRE predictive performance and clinical utility across eGFR equations and biomarkers were similar when restricting to individuals with confirmed CKD (Figs S12**–**S14).

## DISCUSSION

In this cohort study of 27 125 individuals with simultaneous creatinine and cystatin C measurements, we evaluated the impact of using EKFC equations on KFRE performance and clinical utility within a European health system. Across all equations, filtration markers, and prediction horizons, discrimination remained excellent. While calibration of eGFR_cr_ was comparable between EKFC and CKD-EPI, EKFC demonstrated improved calibration over CKD-EPI for eGFR_cys_ and eGFR_cr-cys_. Importantly, KFRE’s clinical benefit was similar for both equation sets at currently recommended thresholds. Taken together, these findings indicate that replacing CKD-EPI with EKFC eGFR equations would have negligible influence on KFRE performance or clinical utility.

KFRE demonstrated excellent discrimination regardless of equation choice. Similar to our study, previous investigations have reported very high discrimination of KFRE risk estimates [4, 26, 27]. This is expected given the exponential relationship between eGFR and KFRT risk, whereby differences in eGFR values spanning 10–60 ml/min/1.73 m² yield strong discrimination between individuals at low and high risk.[28]

Calibration is important for providing patients with accurate information about their individual risks. At 2 years, regardless of filtration marker, EKFC demonstrated excellent calibration and outperformed CKD-EPI when eGFR_cys_ or eGFR_cr-cys_ were used. These differences likely reflect the interplay between filtration marker choice and equation-specific biases in GFR estimation. Because eGFR_cys_ yields systematically lower values than eGFR_cr_ [29], cystatin C-based KFRE predictions tend toward higher estimated risks. When CKD-EPI equations are used, this tendency results in overprediction. However, EKFC produces higher eGFR_cys_ estimates than CKD-EPI, thereby resulting in improved calibration. The overprediction observed at 5 years for both equation sets across all filtration markers likely reflects KFRE’s failure to account for the competing risk of death. KFRE was developed without competing risk modeling, implicitly assuming that patients remained at risk of KFRT even after death. In contrast to earlier studies reporting underprediction [26, 27], we incorporated competing risks to assess the absolute risk of KFRT, a measure more clinically relevant. Because patients who die cannot subsequently experience KFRT, the absolute risk is lower than KFRE’s predictions. This discrepancy becomes more pronounced over longer follow-up periods and in older patients, explaining why overprediction was stronger at 5 years than at 2 years, and in individuals aged ≥75 years compared to those aged <75 years.

The KFRE has been endorsed by international guidelines as a decision-support tool for guiding nephrology referrals, initiation of multidisciplinary care, and preparation for KRT [1]. These decisions involve a trade-off between benefits, such as ensuring appropriate care for patients at risk, and potential harms, including unnecessary medical visits or procedures like arterio-venous fistula creation. For this reason, the guidelines proposed specific thresholds that reflect an acceptable balance between these competing considerations. In our study, KFRE-predicted risks derived from EKFC and CKD-EPI eGFR equations provided similarly high net benefit at the 2-year horizon, underscoring the model’s usefulness for care planning in patients with advanced CKD, for whom short-term risk stratification is most critical. Interestingly, although eGFR_cys_ is a stronger predictor of KFRT than eGFR_cr_ and has been advocated on this basis [30], KFRE offered little, if any, clinical utility at the 5-year horizon when used with eGFR_cys_. This likely reflects KFRE’s suboptimal calibration when used with eGFR_cys_. Additionally, KFRE predictions using eGFR_cr_ or eGFR_cr-cys_ showed similar performance. Given that eGFR_cr_ is recommended as a first-line approach for GFR estimation, whereas eGFR_cr-cys_ is preferred in settings where creatinine may be biased [1], these findings suggest that clinicians may rely on the equation deemed most appropriate for GFR estimation in their specific context for risk prediction as well. This study has several strengths. First, it draws on a large, population-based cohort with virtually complete follow-up and comprehensive capture of KFRT. Second, although our primary cohort required only one eGFR test to be included, we confirmed similar findings in the subpopulation with confirmed CKD. Third, creatinine and cystatin C measurements were standardized, ensuring high analytical consistency. Fourth, we assessed discrimination using time-dependent AUCs rather than the conventional c-index, providing a more appropriate measure of predictive accuracy in survival settings [23]. Finally, we assessed both model performance and clinical utility, allowing for a comprehensive evaluation of the potential impact of using EKFC vs. CKD-EPI equations in the KFRE.

Our study also has limitations. It was conducted among healthcare users in the region of Stockholm, and caution is warranted when generalizing findings to other regions. In addition, we restricted the cohort to individuals with simultaneous creatinine and cystatin C measurements, and an albuminuria measurement within 12 months. Although cystatin C testing is less common in many countries, Sweden has comparatively widespread use of cystatin C in routine clinical practice [31]. Nonetheless, our study population is not a random sample of the broader CKD population, as individuals with cystatin C or albuminuria measured are typically older and have more comorbidities [31, 32]. Also, we did not have information on conservative (non-dialysis) management at baseline; however, given that only 3% of the study population had a CKD-EPI eGFR_cr_ between 10 and 15 ml/min/1.73 m², the proportion of such patients in our cohort was likely low. Finally, we were unable to evaluate the 8-variable KFRE, which, although more accurate than the 4-variable version, is used less frequently in routine care.

## CONCLUSION

This study shows that the predictive performance and clinical utility of KFRE is largely unaffected by the choice of eGFR equation used.

## Supplementary Material

sfag187_Supplemental_Files

## Data Availability

Data will be available for collaborative research under reasonable request and fulfillment of GDPR regulations. For inquiries, please send your proposal to the Steering Committee of the SCREAM project (email: juan.jesus.carrero@ki.se).
