# Supplementary material for: Similar predictive performance and clinical utility of the Kidney Failure Risk Equation using EKFC or CKD-EPI estimated glomerular filtration rate
Source: Clin Kidney J. 2026 Jun 8;19(7):sfag187. doi: 10.1093/ckj/sfag187 (PMC13344174; doi:10.1093/ckj/sfag187)
Supplement: sfag187_Supplemental_Files [file sfag187_supplemental_files.zip › SUPPLEMENTS_KFRE-performance-with-EKFC_CKJ-review1-v3.pdf]

Supplemental methods. 2
Table S1. Formulas used to calculate eGFR and KFRE 4
Table S2. Definition of study covariates 5
Table S3. Summary of model performance metrics and their interpretation 6
Table S4. Outcomes and time until first event of included patients 9
Table S5. Comparison of baseline characteristics between the Non-North American KFRE development cohort and the included patients from the SCREAM cohort 10
Table S6. Brier scores, scaled Brier scores and delta Brier scores for the 2-year and 5-year KFRE predictions using CKD-EPI or EKFC eGFR equations. 11
Table S7. Discrimination of the 2-year and 5-year KFRE predictions using CKD-EPI or EKFC eGFR equations, stratified by sex, age and CKD stage. 12
Figure S1. Study design diagram outlining eligibility criteria, covariate assessment, and follow-up period 13
Figure S2. Flow chart illustrating participant selection and exclusion criteria 14
Figure S3. Density plots showing the distribution of eGFR for the CKD-EPI and EKFC equations 15
Figure S4. Observed-to-Expected (O/E) ratio for the 2-year and 5-year KFRE predictions using CKD-EPI or EKFC eGFR equations 16
Figure S5. Distribution of predicted 2-year and 5-year KFRE risks for CKD-EPI or EKFC eGFR equations 17
Figure S6. Distribution of individual-level changes in KFRE-predicted risk when switching from CKD-EPI to EKFC equations 18
Figure S7. Calibration plots of 2-year and 5-year KFRE using CKD-EPI or EKFC eGFR equations, stratified by sex 19
Figure S8. Calibration plots of 2-year and 5-year KFRE using CKD-EPI or EKFC eGFR equations, stratified by age 20
Figure S9. Calibration plots of 2-year and 5-year KFRE using CKD-EPI or EKFC eGFR equations, stratified by CKD stage 21
Figure S10. Decision curve analysis of 2-year and 5-year KFRE predictions using CKD-EPI or EKFC eGFR equations, stratified by sex 22
Figure S11. Decision curve analysis of 2-year and 5-year KFRE predictions using CKD-EPI or EKFC eGFR equations, stratified by age 23
Figure S12. Discrimination of the 2-year and 5-year KFRE predictions using CKD-EPI or EKFC eGFR equations in individuals with at least two eGFR measurements <60 ml/min/1.73m^2 24
Figure S13. Calibration plots of 2-year and 5-year KFRE using CKD-EPI or EKFC eGFR equations in individuals with at least two eGFR measurements <60 ml/min/1.73m2 25
Figure S14. Decision curve analysis of 2-year and 5-year KFRE predictions using CKD-EPI or EKFC eGFR equations, in individuals with at least two eGFR measurements <60 ml/min/1.73m2 26

Supplemental methods.

Performance metrics for predictive models were originally developed for settings in which all outcomes are fully observed and no competing events occur. In survival analyses, however, some patients are censored (their outcome is not observed) while others may experience competing events, which preclude the outcome of interest. Below, we describe versions of these metrics that enable meaningful assessment of model performance in survival settings with competing risks. Although the specific approaches differ by metric (e.g., inverse probability of censoring weights, pseudo-values), the additional complexities ultimately address the two challenges of censoring and competing risks.

<u>Discrimination</u>

We assessed discrimination using the time-dependent area under the receiver operating characteristic curve (AUC).[1,2] We defined cases as individuals who experienced KFRT before or at time t, and controls as individuals who were alive without KFRT at time t. Loss to follow-up (e.g. due to administrative censoring) before time t was accounted for using inverse probability of censoring weighting. We estimated 95% confidence intervals using the asymptotic normality of estimated influence functions.

<u>Calibration</u>

We evaluated calibration using (i) calibration plots, (ii) calibration intercept and slope, and (iii) the observed-to-expected (O/E) ratio. For (i) and (ii), and to account for censoring and the competing risk of death, observed risks of KFRT were estimated using pseudo-values derived from the non-parametric cumulative incidence function (CIF).[3] Calibration curves were constructed by smoothing the individual-level pseudo-values against predicted risk using locally weighted scatterplot smoothing (LOESS), while the intercept and slope were estimated on the complementary log-log scale with robust standard errors.[3] The O/E ratio was calculated as the ratio of the observed outcome proportion estimated by the CIF, and the average risk estimated by the KFRE.[3] We estimated 95% confidence intervals for calibration plots, intercepts and slopes using the normal approximation, and applied bootstrapping with 500 resamples to derive confidence intervals for the O/E ratios.

<u>Overall accuracy</u>

In the presence of competing risks, the Brier score is the average squared difference between the observed risk at the end of the prediction horizon, obtained using pseudo-values, and the predicted absolute risk estimates by that time point, and is defined as: $Brier(t) = \frac{1}{n}\sum_{i=1}^{n} (\widehat{F}_i(t) - \widetilde{F}_i(t))^2$, where $\hat{F}_i(t)$ is the predicted risk for individual *i* at time t, and $\tilde{F}_i(t)$ is the corresponding pseudo-value.

However, when event risks are low, even a non-informative model (one that predicts the same risk for everyone) will have a small Brier score simply because most individuals do not experience the event. This makes it harder to interpret the absolute value of the Brier score, as lower scores do not always mean better performance. To address this, we also calculated a scaled Brier score: 1 − (model Brier score ÷ null model Brier score). Here, the null model assumes equal risk for all individuals, with risk estimates derived from the Aalen–Johansen estimator. The scaled Brier score quantifies the reduction in prediction error from the null model achieved by using the prediction model, with 100% indicating a perfect model, 0% an ineffective model, and <0% indicating worse performance than the null model. We estimated 95% confidence intervals for Brier and scaled Brier score using the normal approximation and applied bootstrapping with 500 resamples to derive confidence intervals for the delta scaled Brier score.

Clinical utility

Net benefit is defined as the true positive rate minus a discounted false positive rate, with false positives penalized by a factor that reflects the relative harm of unnecessary interventions. For example, when setting the threshold probability at 10%, we strategize to refer patients when the 2-year KFRE model predicts a 2-year risk of KFRT above 10%. If at a threshold of 10%, a net benefit of 0.06 results, this means that using the 2-year KFRE model to guide referral to multidisciplinary kidney care would result in the equivalent of six additional correct referrals for patients who will develop KFRT within two years per 100 patients, without increasing unnecessary referrals, compared with a strategy of referring no patients. The preferred model is the one that yields the greatest net benefit.

Table S1. Formulas used to calculate eGFR and KFRE

CKD-EPI equations	Age	Sex	Serum creatinine (µmol/L) / Serum cystatin C (mg/L)		Equation
**$eGFR_{cr}$ CKD-EPI 2009**	≥18	Female	SCr ≤ 62		144 × (SCr/62)$^{-0.329}$ × (0.993)^{Age}
			SCr > 62		144 × (SCr/62)$^{-1.209}$ × (0.993)^{Age}
		Male	SCr ≤ 80		141 × (SCr/80)$^{-0.411}$ × (0.993)^{Age}
			SCr > 80		141 × (SCr/80)$^{-1.209}$ × (0.993)^{Age}
**$eGFR_{cys}$ CKD-EPI 2012**	≥18	Female	ScysC ≤ 0.8		133 x (SCysC/0.8)$^{-0.499}$ x 0.9962^{Age} x 0.932
			ScysC > 0.8		133 x (SCysC/0.8)$^{-1.328}$ x 0.9962^{Age} x 0.932
		Male	ScysC ≤ 0.8		133 x (SCysC/0.8)$^{-0.499}$ x 0.9962^{Age}
			ScysC > 0.8		133 x (SCysC/0.8)$^{-1.328}$ x 0.9962^{Age}
**$eGFR_{cr\text{-}cys}$ CKD-EPI 2012**	≥ 18	Female	SCr ≤ 62	ScysC ≤ 0.8	130 x (SCr/62)$^{-0.248}$ x (SCysC/0.8)$^{-0.375}$ x 0.9952^{Age}
			SCr > 62	ScysC ≤ 0.8	130 x (SCr/62)$^{-0.601}$ x (SCysC/0.8)$^{-0.375}$ x 0.9952^{Age}
			SCr ≤ 62	ScysC > 0.8	130 x (SCr/62)$^{-0.248}$ x (SCysC/0.8)$^{-0.711}$ x 0.9952^{Age}
			SCr > 62	ScysC > 0.8	130 x (SCr/62)$^{-0.601}$ x (SCysC/0.8)$^{-0.711}$ x 0.9952^{Age}
		Male	SCr ≤ 80	ScysC ≤ 0.8	135 x (SCr/80)$^{-0.207}$ x (SCysC/0.8)$^{-0.375}$ x 0.9952^{Age}
			SCr > 80	ScysC ≤ 0.8	135 x (SCr/80)$^{-0.601}$ x (SCysC/0.8)$^{-0.375}$ x 0.9952^{Age}
			SCr ≤ 80	ScysC > 0.8	135 x (SCr/80)$^{-0.207}$ x (SCysC/0.8)$^{-0.711}$ x 0.9952^{Age}
			SCr > 80	ScysC > 0.8	135 x (SCr/80)$^{-0.601}$ x (SCysC/0.8)$^{-0.711}$ x 0.9952^{Age}

EKFC equations	Age	Sex	Q value
**$eGFR_{cr}$** EKFC	Age ≤25	Male	exp(3.200 + 0.259·age − 0.543·ln(age) − 0.00763·age^2 + 0.0000790·age^3)
		Female	exp(3.080 + 0.177·age − 0.223·ln(age) − 0.00596·age^2 + 0.0000686·age^3)
	age >25	Male	80
		Female	62
**$eGFR_{cys}$** EKFC	age ≤ 50	-	0.83
	age > 50	-	0.83 + 0.005·(age − 50)

EKFC equations	Age	SCr/Q or SCys/Q	Equation
**$eGFR_{cr}$ EKFC**	Age ≤40	SCr/Q <1	107.3·(SCr/Q)$^{-0.322}$
		SCr/Q ≥1	107.3·(SCr/Q)$^{-1.132}$
	Age >40	SCr/Q <1	107.3·(SCr/Q)$^{-0.322}$·0.990^{age-40}
		SCr/Q ≥1	107.3·(SCr/Q)$^{-1.132}$· 0.990^{age-40}
**$eGFR_{cys}$ EKFC**	18 ≤ age ≤ 40	SCys/Q < 1	107.3·(cystatin/Q)$^{-0.322}$
		SCys/Q ≥ 1	107.3·(cystatin/Q)$^{-1.132}$
	age > 40	SCys/Q < 1	107.3·(cystatin/Q)$^{-0.322}$·0.990^{age-40}
		SCys/Q ≥ 1	107.3·(cystatin/Q)$^{-1.132}$·0.990^{age-40}
**$eGFR_{cr\text{-}cys}$ EKFC**	-	-	($EKFC_{cr\,+}$ EKFCcys)/2

**Non-North American KFRE (age in years, eGFR in ml/min/1.73^2, ACR in mg/g):**	
Linear predictor	LP = -0.2201 × (age/10 – 7.036) + 0.2467 × (male – 0.5642) – 0.5567 × (eGFR/5 – 7.222) + 0.4510 × (logACR – 5.137)
2-year KFRE	1 – 0.9832$^{exp(LP)}$
5-year KFRE	1 – 0.9365$^{exp(LP)}$

Table S2. Definition of study covariates

Comorbidities*	ICD-10 codes
Myocardial infarction	I200, I21-I22
Other Ischemic Heart Disease	I201, I208, I209, I24, I25
Hypertension	I10-I15
Heart Failure	I110, I130, I132, I50
Stroke	I60-I64, I693, I698, I694
Other cerebrovascular disease	I65-I69, G450-G453, G458, G459, G46
Arrhythmia	I44-I49
Peripheral vascular disease	I70, I72, I73
Diabetes mellitus	E10-E14
Cancer in previous year	C00-C26, C30-C34, C37-C42, C43-C76, C81-C86, C88-C97
Chronic obstructive pulmonary disease	J44
Liver disease	B18, I982, K70-K77
**Medication****	**ATC codes**
Beta blocker	C07
Calcium channel blocker	C08
Diabetes medications***	A10
Diuretic	C03
ACEi/ARB	C09A-C09D
Lipid lowering drug	C10
NSAID	M01A

Abbreviations: ICD, International Classification of Disease; ATC, Anatomical Therapeutic Chemical; ACEi/ARB, Angiotensin-Converting Enzyme Inhibitors/Angiotensin II Receptor Blockers; NSAID, Nonsteroidal Anti-Inflammatory Drug

* A comorbidity was considered present if there was an ICD-10 code in general practice, outpatient or inpatient care at any time before the index date

** Ongoing medications were defined as dispensation of the drug in the 183 days prior to the index date

*** Includes insulin, insulin analogues, biguanides, sulfonylureas, DPP-4 inhibitors, SGLT-2 inhibitors, GLP-1 receptor agonists, other antidiabetic agents, and combination products

Table S3. Summary of model performance metrics and their interpretation

Metric	What it evaluates	Range	Interpretation
**Discrimination**			
Time-dependent area under the receiver operating characteristic curve (AUC)<br>(**Figure 1**)	**Discrimination**, which refers to how well the KFRE distinguishes between patients who experience KFRT (cases) and those who are alive without KFRT (controls) at a given time horizon.<br>It reflects the KFRE's ability to rank patients correctly, meaning the focus is not on the exact predicted risk values but on whether patients who eventually develop KFRT are consistently assigned higher predicted risks than those who remain event-free.	0.5 to 1	**The higher the better**<br>• 1: perfect discrimination between cases and controls<br>• 0.5: no discrimination beyond chance
**Calibration**			
Calibration plot<br>(**Figure 2**)	**(Moderate*) calibration**, which describes how closely KFRE's predicted risks match the actual observed KFRT risk.**	0 to 1 on each axis	**The closer to the identity line the better**<br>• The identity line (the diagonal going from [0,0] to [1,1]) denotes perfect calibration.<br>• Curves above indicate underestimation of the KFRT risk by the KFRE (observed > predicted)<br>• Curves below indicate overestimation of the KFRT risk by the KFRE (observed < predicted)
Calibration intercept<br>(**Table 2**)	**(Mean) calibration**, which assesses whether the KFRE's average predicted risk aligns with the observed event risk in the dataset.	$-\infty$ to $+\infty$	**The closer to 0 the better**<br>• 0: perfect mean calibration<br>• >0: systematic underestimation of the KFRT risk by the KFRE, average predicted risks are lower than the observed event rate<br>• <0: systematic overestimation of the KFRT risk by the KFRE, average predicted risks are higher than the observed event rate
Observed/Expected ratio<br>(**Figure S4**)		0 to $+\infty$	**The closer to 1 the better**<br>• 1: perfect mean calibration<br>• >1: systematic underestimation of the KFRT risk by the KFRE<br>• <1: systematic overestimation of the KFRT risk by the KFRE

Calibration slope (**Table 2**)	**(Weak) calibration**, which quantifies whether a model is systematically under or over-confident in the risks it assigns.	-∞ to +∞	**The closer to 1 the better**<br>• 1: perfect spread between observed and predicted KFRT risk<br>• >1: predicted risks are too moderate, i.e. too low for individuals at high risk and too high for those at low risk<br>• <1: predicted risks are too extreme, i.e. too high for individuals at high risk and too low for those at low risk
**Overall accuracy**			
Predicted risk distributions among cases and controls (**Figure 3**)	**Overall accuracy**, which is a combination of discrimination and calibration.	0 to 1	Ideally, this figure would show higher predicted risks among cases than among controls, with limited overlap between the two distributions
Brier score (**Table S4**)	**Overall accuracy**, the quadratic difference between KFRE's predicted risks and actual observed KFRT risk	0 to 1	**The lower the better**<br>• 0: perfect overall accuracy<br>• 1: worst overall accuracy
Scaled Brier score (**Table S4**)	**Overall accuracy**, comparing the Brier score of the model with a non-informative model, which assigns the average observed risk (i.e., event rate) of KFRT to all individuals. $Scaled\ Brier = 1 - \frac{Brier_{model}}{Bier_{non-informative}}$	-∞ to 1	**The higher the better**<br>• 1: perfect overall accuracy<br>• 0: no improvement over the non-informative model<br>• Values between 0 and 1: increasing improvement over the non-informative model<br>• Negative values: worse performance than the non-informative model
Delta scaled Brier score (**Table S4**)	**Overall accuracy**, comparing the scaled Brier scores of two models, i.e. how much the new model improves overall prediction accuracy over a non-informative model, compared to the other model. $\Delta Scaled\ Brier = Scaled\ Brier_{model\ 1} - Scaled\ Brier_{model\ 2}$	-1 to 1***	**The higher, the better the new model**<br>• 0: no difference in overall accuracy between the two models<br>• >0: the new model performs better than the reference model<br>• <0: the new model performs worse than the reference model
**Clinical utility**			
Net benefit	**Net benefit** weights the benefit of correctly identifying patients who will experience KFRT against the harm of unnecessary interventions (referral) in patients who will not.	-∞ to the observed proportion of KFRT	**The higher the better**<br>• 0: using this strategy is as beneficial as treating no one<br>• <0: using this strategy is worse than treating no one<br>• >0: using this strategy is better than treating no one

Decision curve analysis (**Figure 4**)	It compares the net benefit of applying different strategies (treating no one, treating everyone, use EKFC to compute KFRE and guide decisions, or use CKD-EPI to compute KFRE and guide decisions)	x-axis: pre-specified range of probabilities y-axis: similar to net benefit	**At a pre-specified range of KFRE threshold probabilities, the strategy having the highest net benefit is the one that is clinically most useful.**

* See Van Calster et al.[4] for an explanation of the different calibration measures.

** Note that a model may demonstrate strong discrimination yet still be poorly calibrated, and the opposite can also be true.

***Assuming that both models perform better than the non-informative one. Abbreviations: AUC, time-dependent area under the receiver operating characteristic curve; KFRT: kidney failure with replacement therapy; KFRE: kidney failure risk equation.

Table S4. Outcomes and time until first event of included patients

Characteristic	N = 27,125
**Outcome 2 years, n (%)***	
Alive without KFRT	22,291 (82.2%)
KFRT	620 (2.3%)
Death without KFRT	4,214 (15.5%)
Median time to event (Q1-Q3), days **	731 (731, 731)
**Outcome 5 years, n (%)***	
Alive without KFRT	17,573 (64.8%)
KFRT	1,265 (4.7%)
Death without KFRT	8,287 (30.6%)
Median time to event (Q1-Q3), days **	1,558 (967, 1,826)

*Risks for each outcome were estimated using the cumulative incidence function (CIF) to account for competing risks

** Time from baseline to the first occurrence of KFRT, death, or end of follow-up

Abbreviations: KFRT, kidney failure with replacement therapy.

Table S5. Comparison of baseline characteristics between the Non-North American KFRE development cohort and the included patients from the SCREAM cohort

	Non-north American KFRE (N = 721,357)	SCREAM (N = 27,125)
Age, mean (SD)	74 (10)	73 (13)
Men, n (%)	553,383 (77%)	14,986 (55%)
eGFR, mean (SD)	46 (11)	42 (13)
KFRT events, n (%)	23,829 (3.3%)	1,265 (4.7%)

Abbreviations: eGFR, estimated glomerular filtration rate (creatinine-based CKD-EPI 2009); KFRT, Kidney Failure with Replacement Therapy

Table S6. Brier scores, scaled Brier scores and delta Brier scores for the 2-year and 5-year KFRE predictions using CKD-EPI or EKFC eGFR equations.

	2-year KFRE			5-year KFRE		
**Equation**	**Brier Score**	**Scaled Brier Score**	**Delta Scaled Brier**	**Brier Score**	**Scaled Brier Score**	**Delta Scaled Brier**
**$eGFR_{cr}$**						
CKD-EPI	0.01 (0.01; 0.01)	0.43 (0.40; 0.45)	-	0.03 (0.03; 0.03)	0.41 (0.38; 0.44)	-
EKFC	0.01 (0.01; 0.01)	0.40 (0.38; 0.43)	-2.23% (-2.46; -2.00)	0.03 (0.03; 0.03)	0.38 (0.35; 0.42)	-2.10% (-2.31; -1.91)
**$eGFR_{cys}$**						
CKD-EPI	0.01 (0.01; 0.01)	0.40 (0.38; 0.43)	-	0.04 (0.03; 0.04)	0.22 (0.18; 0.26)	-
EKFC	0.01 (0.01; 0.02)	0.34 (0.31; 0.36)	-6.63% (-7.34; -5.84)	0.03 (0.03; 0.03)	0.30 (0.26; 0.33)	7.63% (6.33; 9.09)
**$eGFR_{cr-cys}$**						
CKD-EPI	0.01 (0.01; 0.01)	0.44 (0.41; 0.47)	-	0.03 (0.03; 0.03)	0.34 (0.31; 0.38)	-
EKFC	0.01 (0.01; 0.01)	0.37 (0.35; 0.40)	-6.64% (-7.17; -6.06)	0.03 (0.03; 0.03)	0.36 (0.33; 0.39)	1.95% (1.03; 2.80)

Lower Brier scores and higher scaled Brier scores indicate better predictive performance. The delta scaled Brier score represents the difference in scaled Brier score between EKFC and the reference model (CKD-EPI) for the same filtration marker and prediction horizon. Negative values indicate worse performance of EKFC relative to CKD-EPI, whereas positive values indicate improved performance. Estimates are shown with 95% confidence intervals. Abbreviations: eGFR: estimated glomerular filtration rate; EKFC: European Kidney Function Consortium; CKD-EPI: Chronic Kidney Disease Epidemiology Collaboration 2009-2012; KFRE: kidney failure risk equation.

Table S7. Discrimination of the 2-year and 5-year KFRE predictions using CKD-EPI or EKFC eGFR equations, stratified by sex, age and CKD stage.

	Equation	Sex		Age		CKD stage	
		**Female** (n=12,139)	**Male** (n=14,986)	**<75 years** (n=14,246)	**≥75 years** (n=12,879)	**G3a-3b** (n=21,847)	**G4-5** (n=5,278)
**2-year KFRE**							
$eGFR_{cr}$	CKD-EPI	0.967 (0.977 - 0.957)	0.968 (0.975 - 0.962)	0.965 (0.971 - 0.959)	0.965 (0.980 - 0.951)	0.923 (0.960 - 0.886)	0.882 (0.897 - 0.867)
	EKFC	0.965 (0.976 - 0.955)	0.968 (0.974 - 0.961)	0.965 (0.971 - 0.959)	0.964 (0.979 - 0.949)	0.920 (0.959 - 0.881)	0.879 (0.894 - 0.864)
$eGFR_{cys}$	CKD-EPI	0.963 (0.976 - 0.951)	0.965 (0.971 - 0.959)	0.963 (0.969 - 0.957)	0.957 (0.975 - 0.939)	0.915 (0.958 - 0.871)	0.875 (0.890 - 0.861)
	EKFC	0.963 (0.976 - 0.950)	0.965 (0.971 - 0.959)	0.962 (0.969 - 0.956)	0.958 (0.975 - 0.941)	0.913 (0.956 - 0.869)	0.873 (0.888 - 0.859)
$eGFR_{cr\text{-}cys}$	CKD-EPI	0.968 (0.979 - 0.957)	0.970 (0.976 - 0.965)	0.967 (0.973 - 0.962)	0.965 (0.981 - 0.949)	0.929 (0.967 - 0.891)	0.886 (0.900 - 0.872)
	EKFC	0.967 (0.978 - 0.955)	0.970 (0.976 - 0.964)	0.967 (0.973 - 0.961)	0.965 (0.980 - 0.950)	0.926 (0.964 - 0.887)	0.883 (0.898 - 0.869)
**5-year KFRE**							
$eGFR_{cr}$	CKD-EPI	0.963 (0.971 - 0.955)	0.963 (0.969 - 0.957)	0.960 (0.965 - 0.954)	0.968 (0.978 - 0.958)	0.922 (0.940 - 0.903)	0.885 (0.899 - 0.871)
	EKFC	0.961 (0.970 - 0.952)	0.962 (0.968 - 0.955)	0.959 (0.965 - 0.953)	0.968 (0.978 - 0.957)	0.915 (0.935 - 0.895)	0.882 (0.896 - 0.868)
$eGFR_{cys}$	CKD-EPI	0.954 (0.963 - 0.945)	0.963 (0.968 - 0.957)	0.957 (0.962 - 0.951)	0.963 (0.976 - 0.951)	0.923 (0.940 - 0.905)	0.870 (0.885 - 0.855)
	EKFC	0.953 (0.962 - 0.943)	0.962 (0.968 - 0.956)	0.956 (0.961 - 0.950)	0.964 (0.976 - 0.952)	0.921 (0.939 - 0.903)	0.868 (0.883 - 0.853)
$eGFR_{cr\text{-}cys}$	CKD-EPI	0.964 (0.972 - 0.956)	0.968 (0.973 - 0.962)	0.963 (0.968 - 0.958)	0.969 (0.980 - 0.959)	0.935 (0.951 - 0.919)	0.885 (0.898 - 0.871)
	EKFC	0.962 (0.970 - 0.953)	0.967 (0.972 - 0.961)	0.962 (0.967 - 0.957)	0.970 (0.980 - 0.960)	0.930 (0.947 - 0.913)	0.882 (0.896 - 0.868)

Discrimination was assessed by the time-dependent Area Under the Receiver Operating Characteristics Curve (AUC), with higher values indicating better ability to distinguish between individuals who did and did not progress to KFRT at the prediction horizon.

Abbreviations: eGFR: estimated glomerular filtration rate; KFRE: kidney failure risk equation; EKFC: European Kidney Function Consortium; CKD-EPI: Chronic Kidney Disease Epidemiology Collaboration 2009-2012; CKD: Chronic Kidney Disease.

Figure S1. Study design diagram outlining eligibility criteria, covariate assessment, and follow-up period

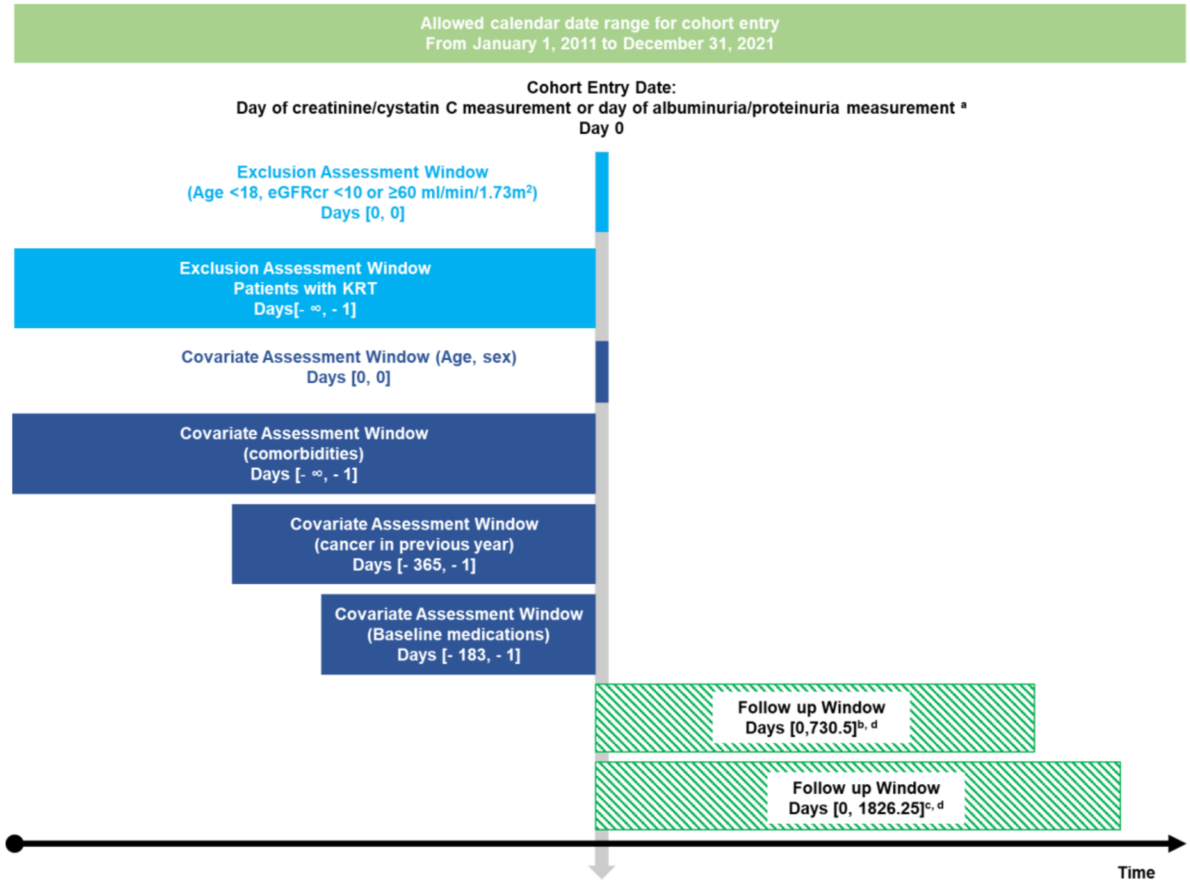

We identified all creatinine and cystatin C measurements taken on the same day within the allowed range for cohort entry. Of these measurements, we only kept the ones that had an albuminuria/proteinuria measurement within the 12 months before or after. We then selected the albuminuria/proteinuria measurement closest to each creatinine/cystatin C measurement. After applying the additional eligibility criteria (age, eGFR, history of KFRT), patients could have multiple eligible measurement pairs (creatinine/cystatin C and albuminuria/proteinuria). In these cases, we chose one eligible measurement pair at random. We subsequently defined the index date as the latest date among the selected creatinine/cystatin C and albuminuria/proteinuria measurement.

a. For the 2-year KFRE
b. For the 5-year KFRE
c. Earliest of: KFRT (outcome of interest), death (competing event), end of prediction horizon (2-years or 5-years), end of study period or emigration from Stockholm region

Abbreviations: eGFR: estimated glomerular filtration rate; KFRT, kidney failure with replacement therapy.

Figure S2. Flow chart illustrating participant selection and exclusion criteria

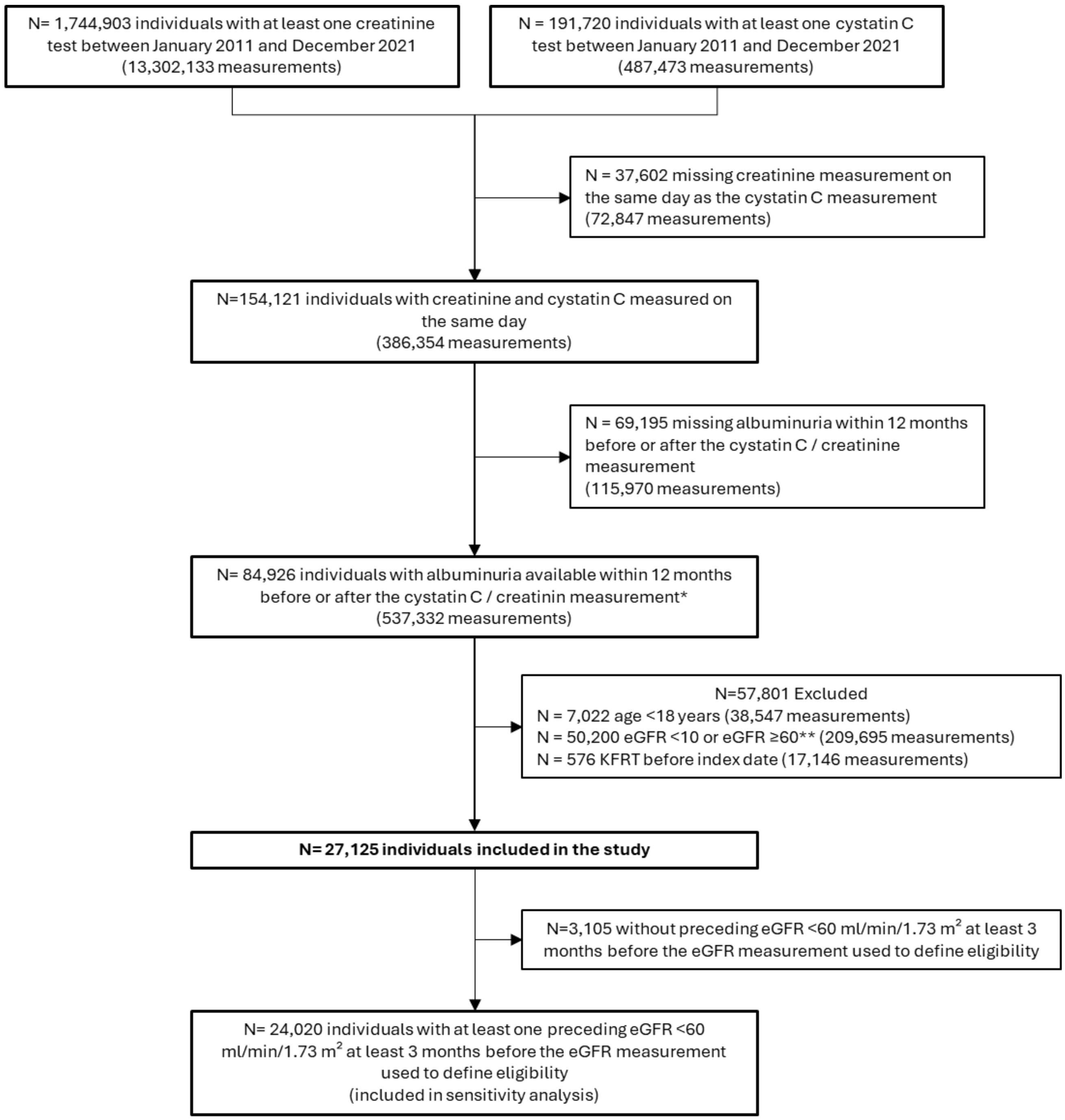

* The median time difference between albuminuria and baseline measurements was 0 days (IQR −45 to 0 days)

** Calculated with the CKD-EPI$_{cr}$2009

Abbreviations: eGFR: estimated glomerular filtration rate; KFRT, kidney failure with replacement therapy.

Figure S3. Density plots showing the distribution of eGFR for the CKD-EPI and EKFC equations

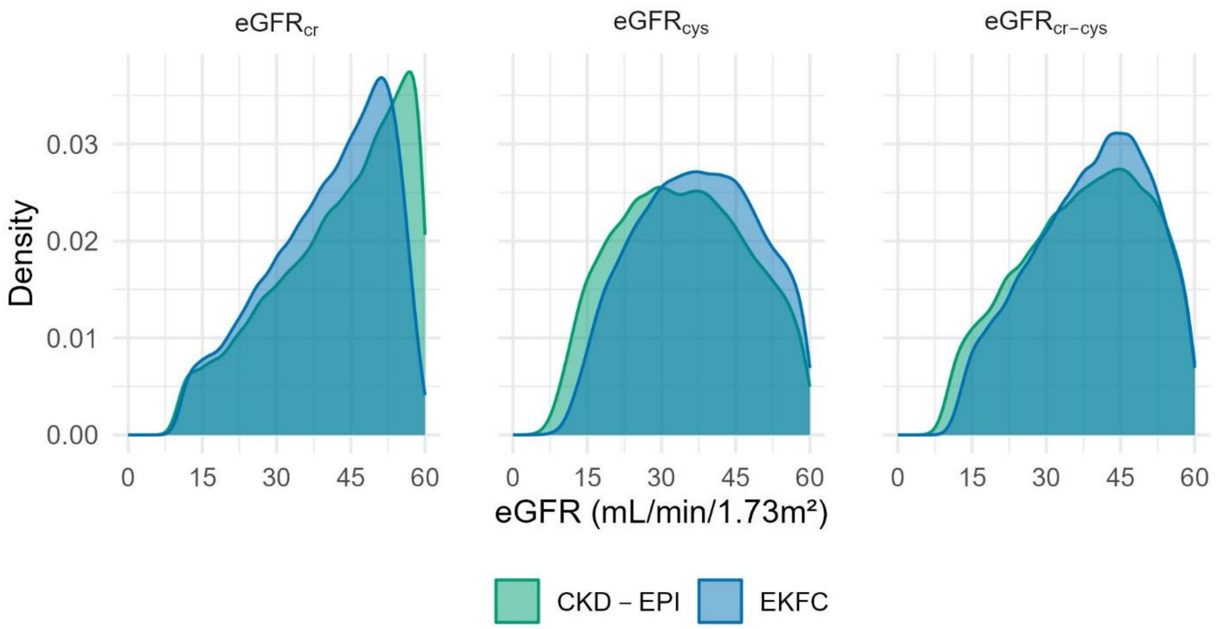

Abbreviations: eGFR: estimated glomerular filtration rate; EKFC: European Kidney Function Consortium; CKD-EPI: Chronic Kidney Disease Epidemiology Collaboration 2009-2012

Figure S4. Observed-to-Expected (O/E) ratio for the 2-year and 5-year KFRE predictions using CKD-EPI or EKFC eGFR equations

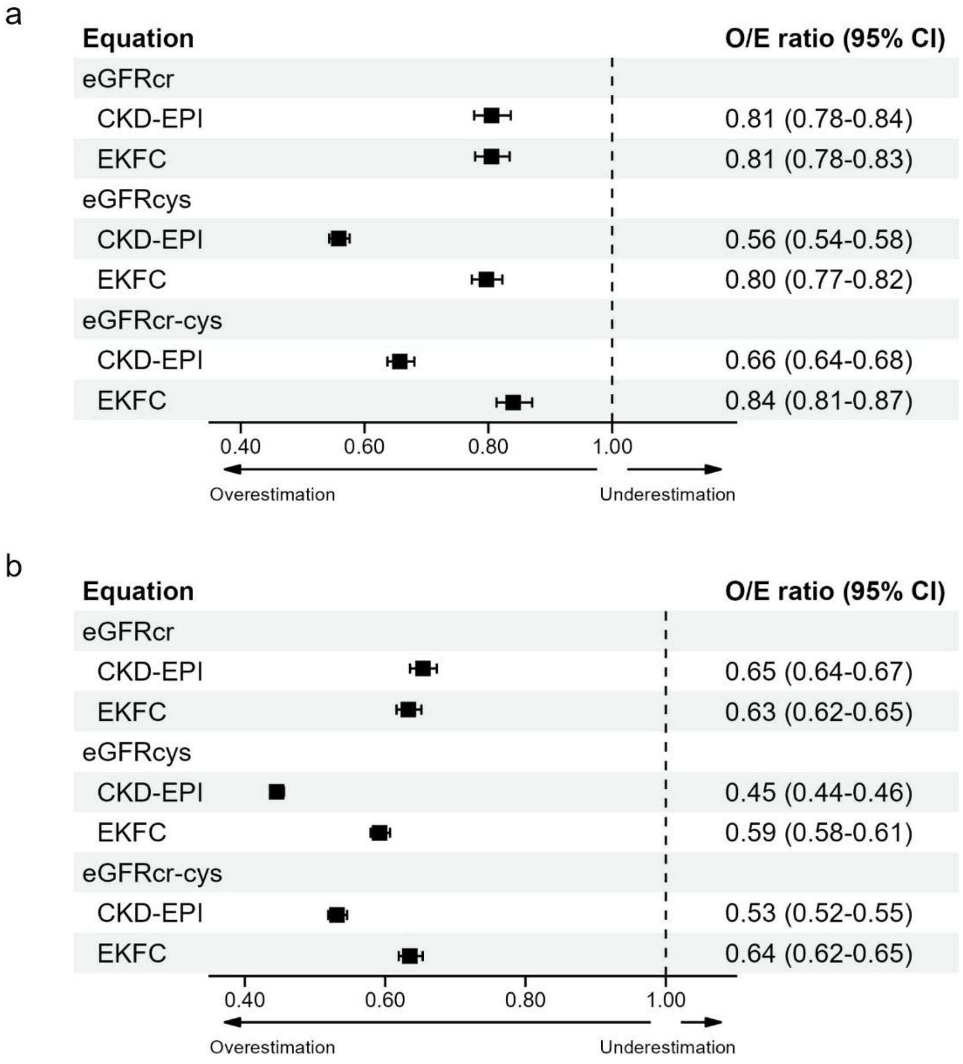

An O/E ratio of 1 indicates perfect average calibration, values >1 indicate underestimation of risk, and values <1 indicate overestimation. Panel (a) represents the 2-year O/E ratio and panel (b) the 5-year O/E ratio, for the 2-year and 5-year KFRE predictions, respectively. Error bars represent 95% confidence intervals and the dashed vertical reference line corresponds to O/E = 1 (perfect calibration).

Abbreviations: eGFR: estimated glomerular filtration rate; KFRE: kidney failure risk equation; EKFC: European Kidney Function Consortium; CKD-EPI: Chronic Kidney Disease Epidemiology Collaboration 2009-2012; O/E, observed-to-expected ratio.

Figure S5. Distribution of predicted 2-year and 5-year KFRE risks for CKD-EPI or EKFC eGFR equations

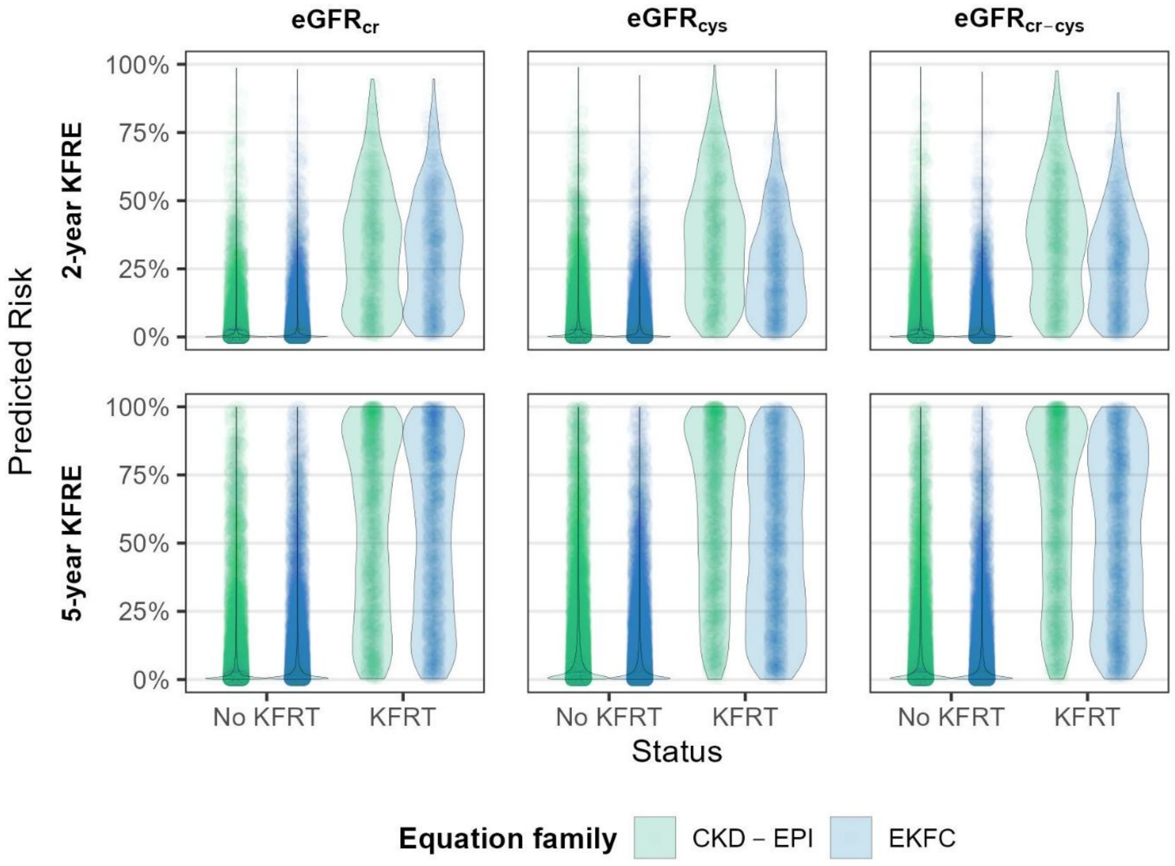

Predicted risks (y-axis) are shown for individuals who did not develop KFRT or died (No KFRT) and those who progressed to kidney failure requiring replacement therapy (KFRT), stratified by equation family and filtration marker. Each panel displays the distribution of predicted risk percentages for CKD-EPI (green) and EKFC (blue) equations. Wider sections of the violin indicate higher density of predictions at that risk level. Panels are organized by prediction horizon (2-year top row, 5-year bottom row) and eGFR estimation method: creatinine-based (left column), cystatin C–based (middle column), and combined creatinine–cystatin C (right column).

Figure S6. Distribution of individual-level changes in KFRE-predicted risk when switching from CKD-EPI to EKFC equations

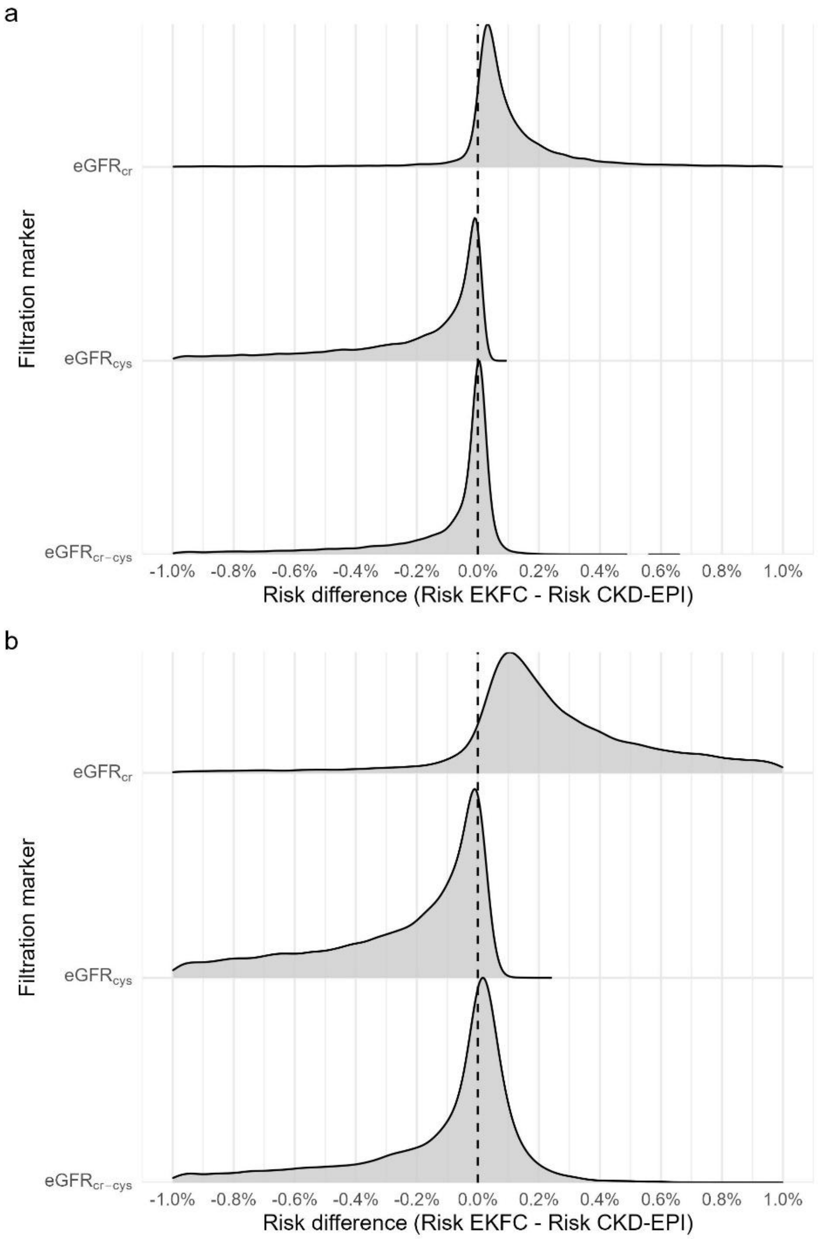

Density plots show the differences in predicted (a) 2-year and (b) 5-year kidney failure risks, for equations based on creatinine ($eGFR_{cr}$), cystatin C ($eGFR_{cys}$), and both markers combined ($eGFR_{cr\text{-}cys}$). The vertical dashed line indicates zero difference. Positive values reflect higher risk estimates with EKFC; negative values reflect lower estimates.

Abbreviations: eGFR: estimated glomerular filtration rate; KFRE: kidney failure risk equation; EKFC: European Kidney Function Consortium; CKD-EPI: Chronic Kidney Disease Epidemiology Collaboration 2009-2012

Figure S7. Calibration plots of 2-year and 5-year KFRE using CKD-EPI or EKFC eGFR equations, stratified by sex

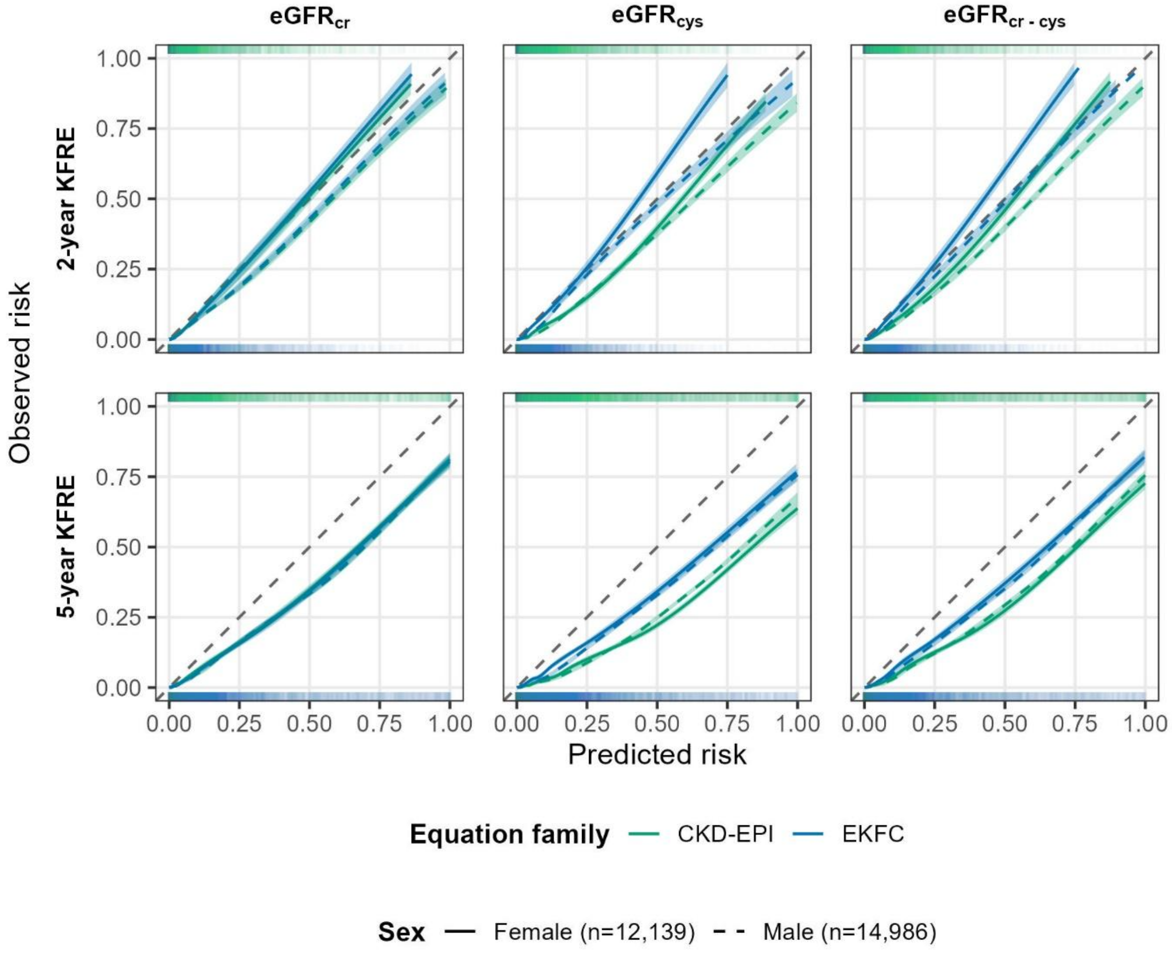

Observed risks (y-axis) are plotted against predicted risks (x-axis) for each equation and filtration marker combination. The solid lines represent loess-smoothed calibration curves, and the dashed diagonal line denotes perfect calibration (observed = predicted). Rug plots along the axes illustrate the distribution of predicted risks. Shaded areas represent 95% confidence intervals. Panels are organized by prediction horizon (2-year top row, 5-year bottom row) and eGFR estimation method: creatinine-based (left column), cystatin C–based (middle column), and combined creatinine–cystatin C (right column).

Abbreviations: eGFR: estimated glomerular filtration rate; KFRE: kidney failure risk equation; EKFC: European Kidney Function Consortium; CKD-EPI: Chronic Kidney Disease Epidemiology Collaboration 2009-2012

Figure S8. Calibration plots of 2-year and 5-year KFRE using CKD-EPI or EKFC eGFR equations, stratified by age

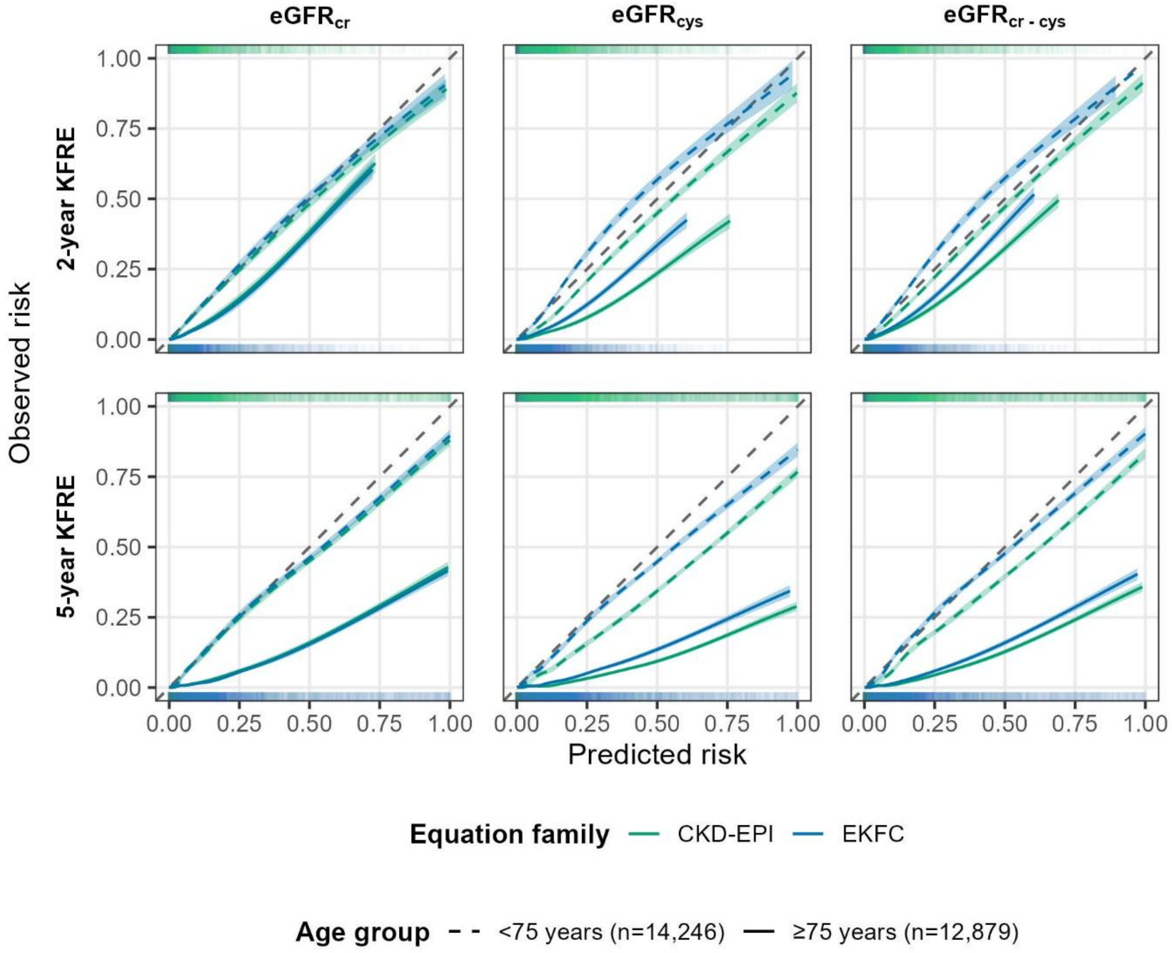

Observed risks (y-axis) are plotted against predicted risks (x-axis) for each equation and filtration marker combination. The solid lines represent loess-smoothed calibration curves, and the dashed diagonal line denotes perfect calibration (observed = predicted). Rug plots along the axes illustrate the distribution of predicted risks. Shaded areas represent 95% confidence intervals. Panels are organized by prediction horizon (2-year top row, 5-year bottom row) and eGFR estimation method: creatinine-based (left column), cystatin C–based (middle column), and combined creatinine–cystatin C (right column).

Abbreviations: eGFR: estimated glomerular filtration rate; KFRE: kidney failure risk equation; EKFC: European Kidney Function Consortium; CKD-EPI: Chronic Kidney Disease Epidemiology Collaboration 2009-2012

Figure S9. Calibration plots of 2-year and 5-year KFRE using CKD-EPI or EKFC eGFR equations, stratified by CKD stage

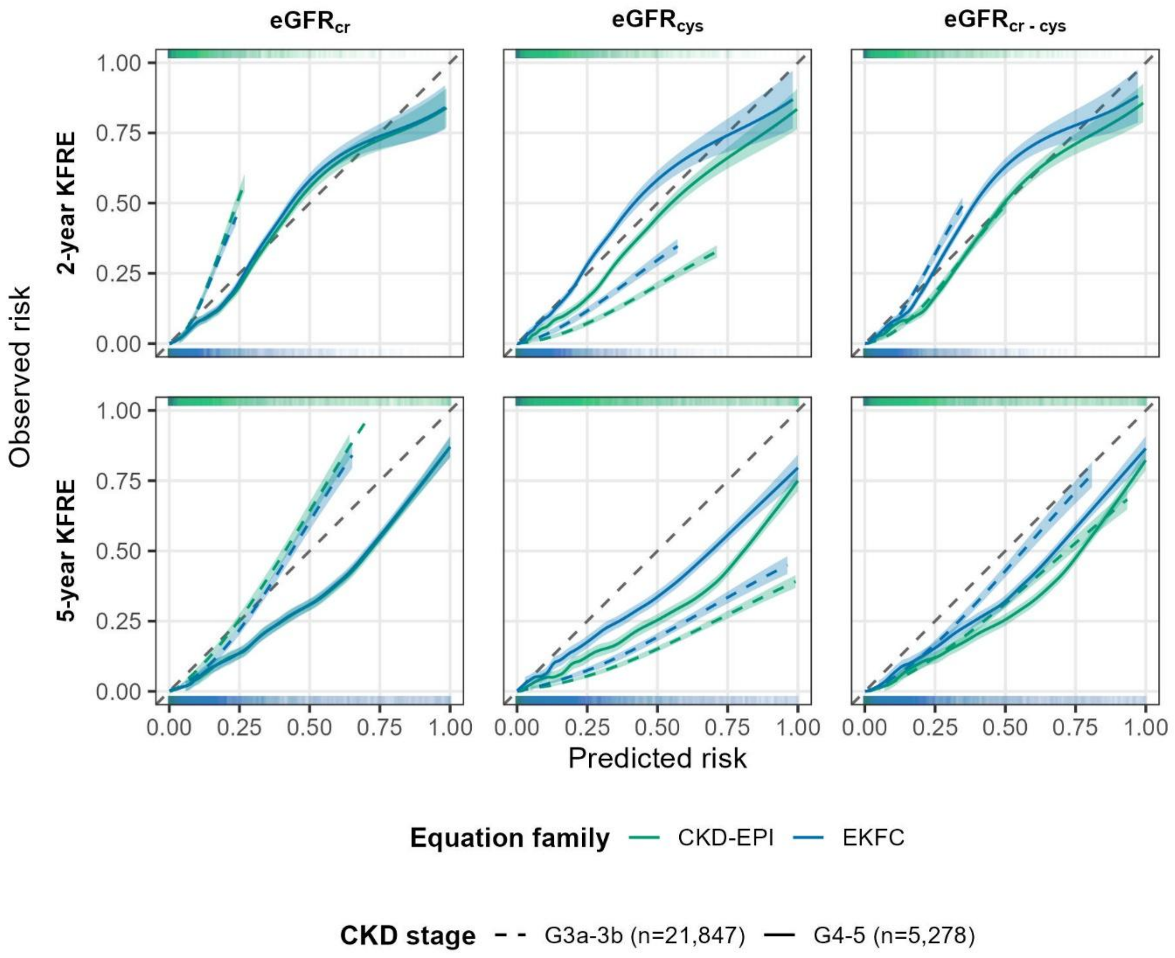

Observed risks (y-axis) are plotted against predicted risks (x-axis) for each equation and filtration marker combination. The solid lines represent loess-smoothed calibration curves, and the dashed diagonal line denotes perfect calibration (observed = predicted). Rug plots along the axes illustrate the distribution of predicted risks. Shaded areas represent 95% confidence intervals. Panels are organized by prediction horizon (2-year top row, 5-year bottom row) and eGFR estimation method: creatinine-based (left column), cystatin C–based (middle column), and combined creatinine–cystatin C (right column).

Abbreviations: eGFR: estimated glomerular filtration rate; KFRE: kidney failure risk equation; EKFC: European Kidney Function Consortium; CKD-EPI: Chronic Kidney Disease Epidemiology Collaboration 2009-2012

Figure S10. Decision curve analysis of 2-year and 5-year KFRE predictions using CKD-EPI or EKFC eGFR equations, stratified by sex

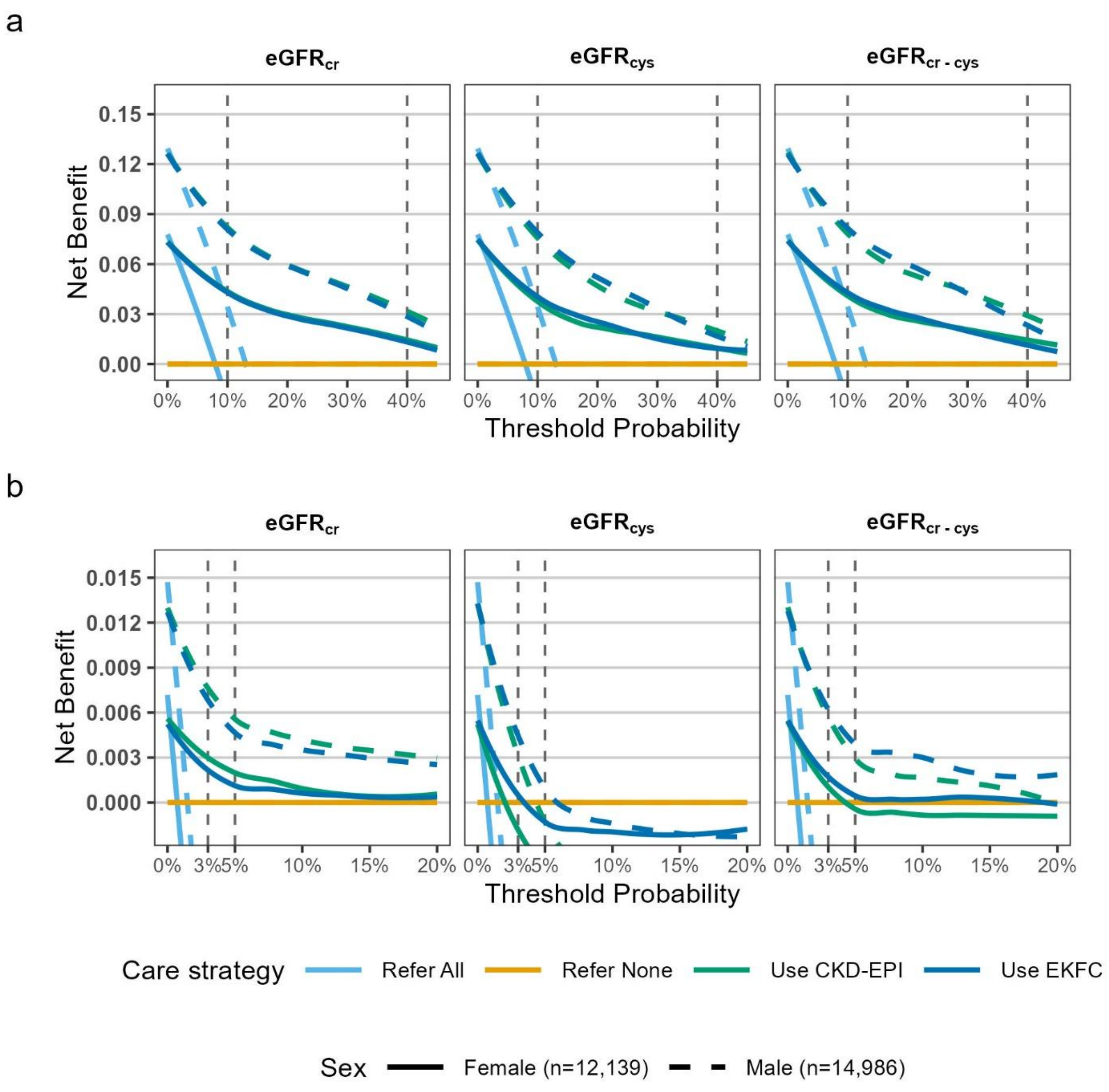

Panel (a) shows the 2-year KFRE in the subset of patients with eGFRcr CKD-EPI 10–29 mL/min/1.73m^2, and panel (b) shows the 5-year KFRE in the subset of patients with $eGFR_{cr}$ CKD-EPI 30-59 mL/min/1.73m^2. Net benefit is plotted across a range of threshold probabilities, with higher curves indicating greater clinical utility. The vertical dashed lines represent the currently recommended thresholds to guide nephrology referral (5-year KFRE of 3-5%), initiation of multidisciplinary care (2-year KFRE of 10%) and preparation for kidney replacement therapy (2-year KFRE of 40%). The "refer all" and "refer none" strategies are included as references. Note that the y-axis scale in panel (b) is 10-fold smaller than in panel (a).

Abbreviations: eGFR: estimated glomerular filtration rate; KFRE: kidney failure risk equation; EKFC: European Kidney Function Consortium; CKD-EPI: Chronic Kidney Disease Epidemiology Collaboration 2009-2012

Figure S11. Decision curve analysis of 2-year and 5-year KFRE predictions using CKD-EPI or EKFC eGFR equations, stratified by age

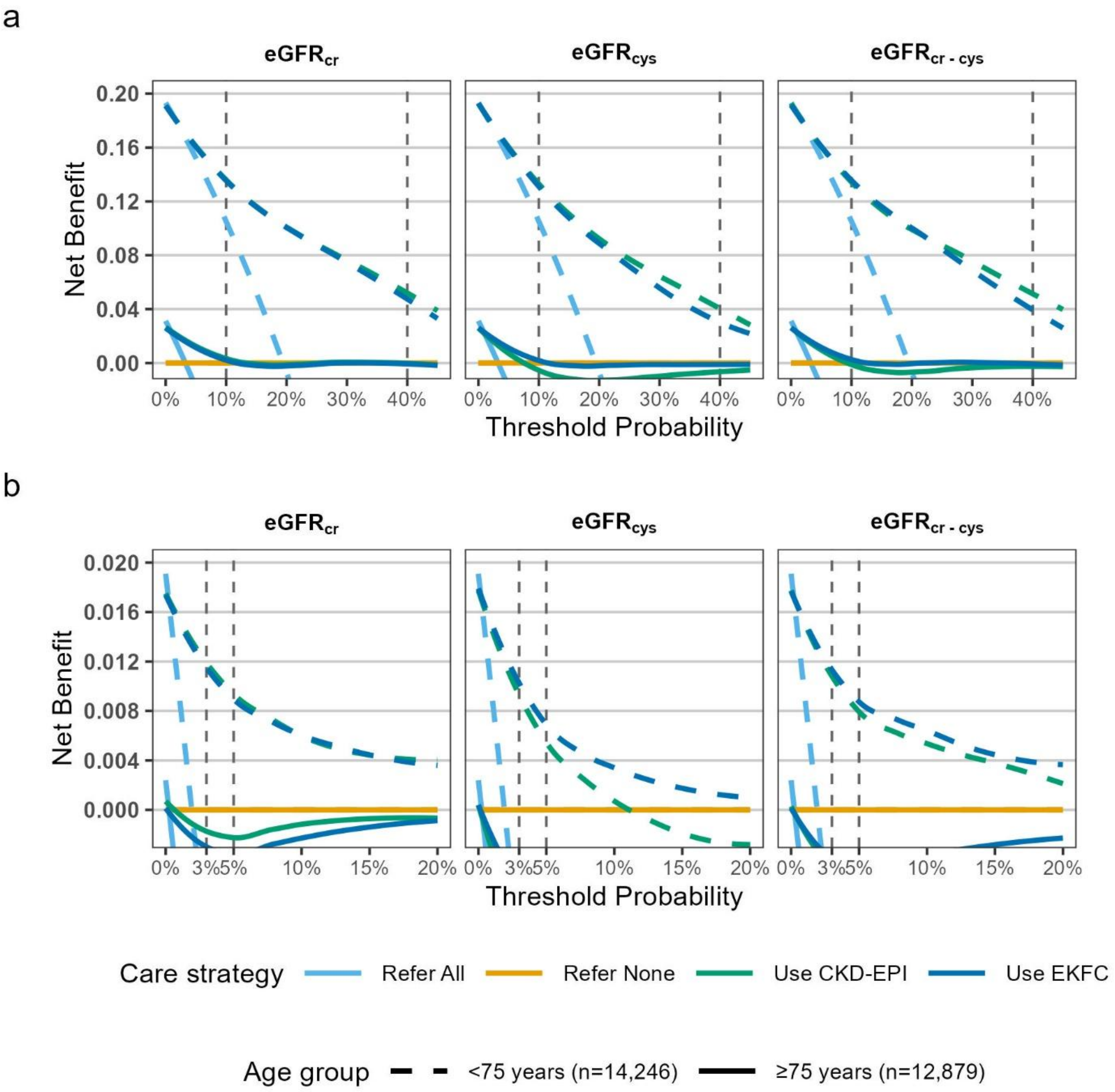

Panel (a) shows the 2-year KFRE in the subset of patients with eGFRcr CKD-EPI 10–29 mL/min/1.73m^2, and panel (b) shows the 5-year KFRE in the subset of patients with $eGFR_{cr}$ CKD-EPI 30-59 mL/min/1.73m^2. Net benefit is plotted across a range of threshold probabilities, with higher curves indicating greater clinical utility. The vertical dashed lines represent the currently recommended thresholds to guide nephrology referral (5-year KFRE of 3-5%), initiation of multidisciplinary care (2-year KFRE of 10%) and preparation for kidney replacement therapy (2-year KFRE of 40%). The “refer all” and “refer none” strategies are included as references. Note that the y-axis scale in panel (b) is 10-fold smaller than in panel (a).

Abbreviations: eGFR: estimated glomerular filtration rate; KFRE: kidney failure risk equation; EKFC: European Kidney Function Consortium; CKD-EPI: Chronic Kidney Disease Epidemiology Collaboration 2009-2012

Figure S12. Discrimination of the 2-year and 5-year KFRE predictions using CKD-EPI or EKFC eGFR equations in individuals with at least two eGFR measurements <60 ml/min/1.73m²

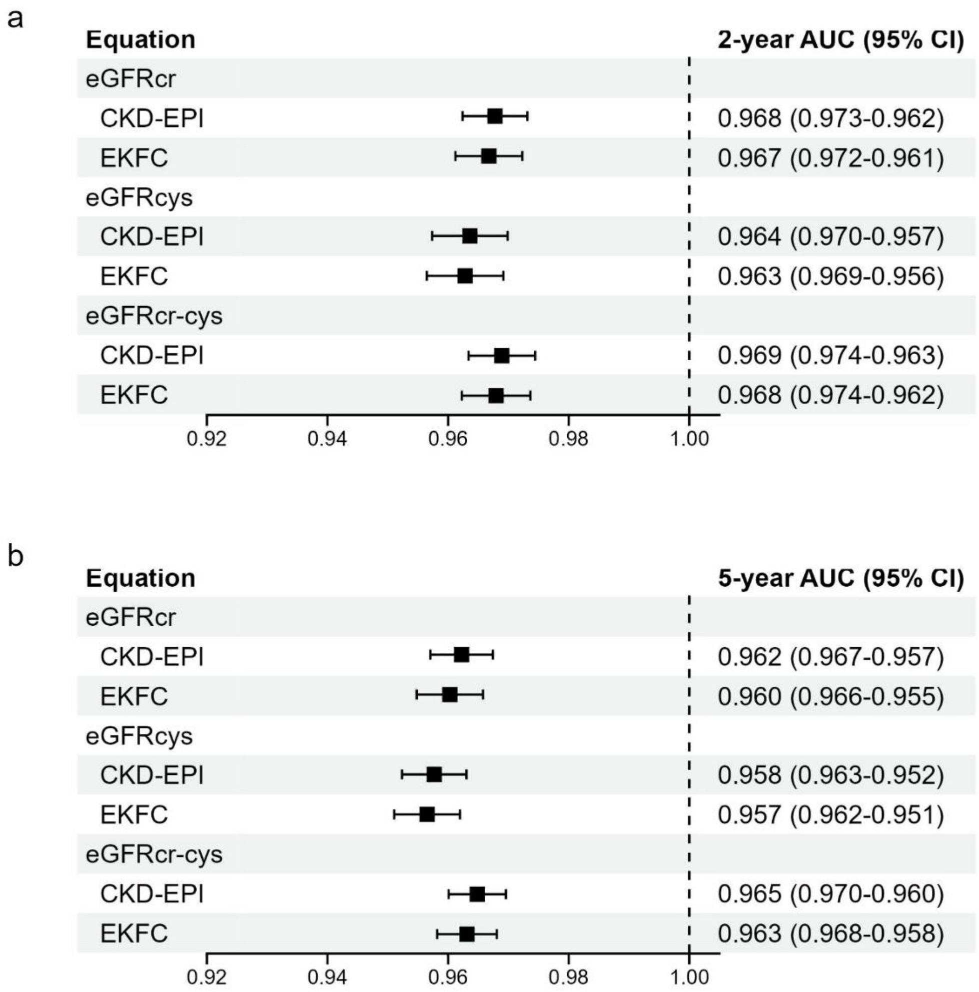

The study population was restricted to individuals with at least one $eGFR_{cr}$ <60 ml/min/1.73m² before study inclusion, as calculated by the CKD-EPI 2009 equation (n=24,020). Discrimination was assessed by the time-dependent Area Under the Receiver Operating Characteristics Curve (AUC), with higher values indicating better ability to distinguish between individuals who did and did not progress to kidney failure at the prediction horizon. Panel (a) represents the 2-year AUC and panel (b) the 5-year AUC, for the 2-year and 5-year KFRE predictions, respectively. Error bars represent 95% confidence intervals.

Figure S13. Calibration plots of 2-year and 5-year KFRE using CKD-EPI or EKFC eGFR equations in individuals with at least two eGFR measurements <60 ml/min/1.73m2

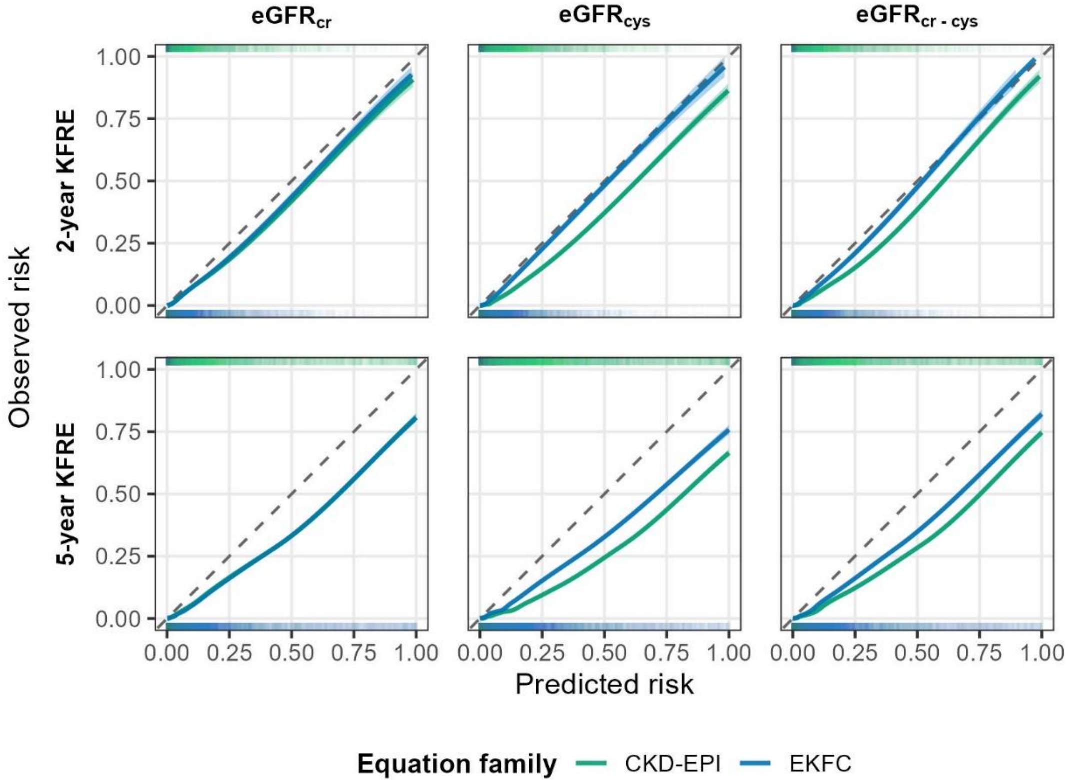

The study population was restricted to individuals with at least one $eGFR_{cr}$ <60 ml/min/1.73m^2 before study inclusion, as calculated by the CKD-EPI 2009 equation (n=24,020). Observed risks (y-axis) are plotted against predicted risks (x-axis) for each equation and filtration marker combination. The solid lines represent loess-smoothed calibration curves, and the dashed diagonal line denotes perfect calibration (observed = predicted). Rug plots along the axes illustrate the distribution of predicted risks. Shaded areas represent 95% confidence intervals. Panels are organized by prediction horizon (2-year top row, 5-year bottom row) and eGFR estimation method: creatinine-based (left column), cystatin C–based (middle column), and combined creatinine–cystatin C (right column).

Abbreviations: eGFR: estimated glomerular filtration rate; KFRE: kidney failure risk equation; EKFC: European Kidney Function Consortium; CKD-EPI: Chronic Kidney Disease Epidemiology Collaboration 2009-2012

Figure S14. Decision curve analysis of 2-year and 5-year KFRE predictions using CKD-EPI or EKFC eGFR equations, in individuals with at least two eGFR measurements <60 ml/min/1.73m2

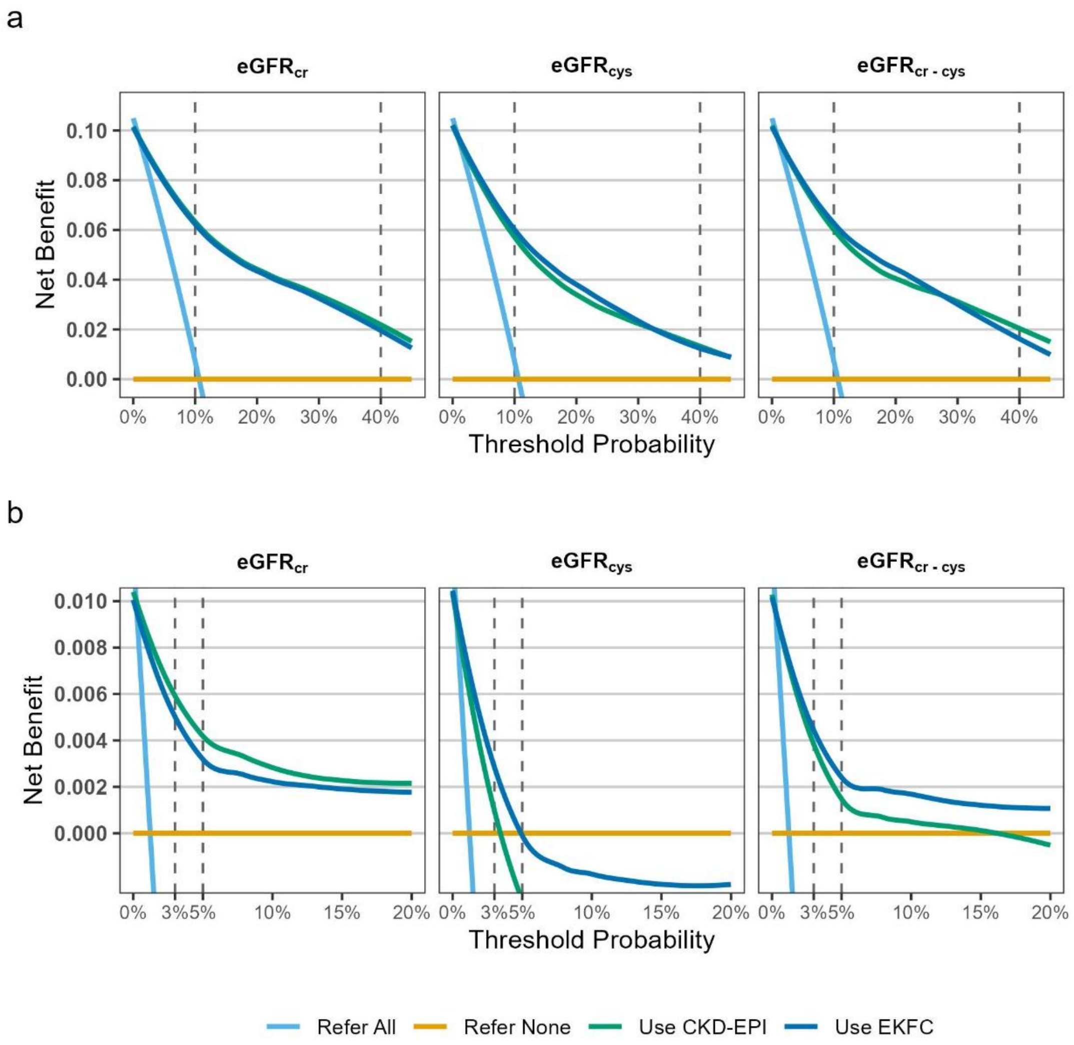

The study population was restricted to individuals with at least one $eGFR_{cr}$ <60 ml/min/1.73m^2 before study inclusion, as calculated by the CKD-EPI 2009 equation (n=24,020). Panel (a) shows the 2-year KFRE in the subset of patients with eGFRcr CKD-EPI 10–29 mL/min/1.73m^2, and panel (b) shows the 5-year KFRE in the subset of patients with $eGFR_{cr}$ CKD-EPI 30-59 mL/min/1.73m^2. Net benefit is plotted across a range of threshold probabilities, with higher curves indicating greater clinical utility. The vertical dashed lines represent the currently recommended thresholds to guide nephrology referral (5-year KFRE of 3-5%), initiation of multidisciplinary care (2-year KFRE of 10%) and preparation for kidney replacement therapy (2-year KFRE of 40%). The “refer all” and “refer none” strategies are included as references. Note that the y-axis scale in panel (b) is 10-fold smaller than in panel (a).

Abbreviations: eGFR: estimated glomerular filtration rate; KFRE: kidney failure risk equation; EKFC: European Kidney Function Consortium; CKD-EPI: Chronic Kidney Disease Epidemiology Collaboration 2009-2012

References

1. Blanche P, Dartigues JF, Jacqmin-Gadda H. Estimating and comparing time-dependent areas under receiver operating characteristic curves for censored event times with competing risks. Stat Med. 2013;32(30):5381-5397. doi:10.1002/sim.5958

2. Blanche P, Kattan MW, Gerds TA. The c-index is not proper for the evaluation of t-year predicted risks. Biostatistics. 2019;20(2):347-357. doi:10.1093/biostatistics/kxy006

3. Van Geloven N, Giardiello D, Bonneville EF, et al. Validation of prediction models in the presence of competing risks: a guide through modern methods. BMJ. 2022;377:e069249. doi:10.1136/bmj-2021-069249

4. Van Calster B, Collins GS, Vickers AJ, et al. Evaluation of performance measures in predictive artificial intelligence models to support medical decisions: overview and guidance. Lancet Digit Health. 2025;7(12):100916. doi:10.1016/j.landig.2025.100916
